# Fully Atomistic Modeling in Computational Spectroscopy: Tryptophan in Aqueous Solution as a Test Case

**DOI:** 10.1021/acs.jpca.5c04511

**Published:** 2025-10-30

**Authors:** Marco Trinari, Chiara Sepali, Tommaso Giovannini, Chiara Cappelli

**Affiliations:** † 226478 Scuola Normale Superiore, Piazza Dei Cavalieri 7, Pisa I-56126, Italy; ‡ Department of Physics, 9318 University of Rome Tor Vergata, and INFN, Via della Ricerca Scientifica 1, Rome I-00133, Italy

## Abstract

We present a multiscale computational protocol for the simulation of a wide range of spectroscopic properties—electronic, magnetic, and vibrational—of zwitterionic l-tryptophan in aqueous solution. The approach combines density functional theory (DFT) for the solute with polarizable embedding models (QM/FQ and QM/FQFμ) for the solvent, and incorporates extensive conformational sampling via classical molecular dynamics. The protocol successfully reproduces UV–vis and ECD spectra, including the characteristic S_0_ → S_1_ transition and chiroptical features, and captures the negative optical rotation at the sodium D-line with good agreement to experiment. NMR chemical shifts and spin–spin couplings are also computed, and a hybrid QM/FDE/FQFμ scheme is employed to improve the description of solvent-sensitive nuclei. Vibrational spectra (IR, Raman, and ROA) are calculated and analyzed, with all models yielding results consistent with experimental data where available. The comparison between QM/FQ and QM/FQFμ highlights the importance of accurate solvent treatment, especially for chiroptical and magnetic properties.

## Graphical abstract

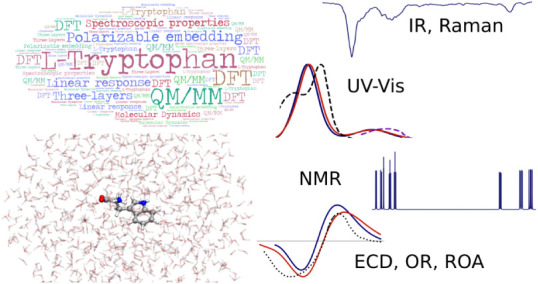

## 1 Introduction

Since its foundations, the field of computational chemistry has expanded in scope, and thanks to both technological and methodological advances, it has become an indispensable tool in most other areas of chemical research.^1^ Among its many applications, a particularly important one is the development of computational methods for the modeling of spectroscopic properties of molecular systems.^2^ Spectroscopic techniques provide direct access to molecular-level information, offering invaluable insights into structure, dynamics, and chemical interactions. As a result, computational spectroscopy has evolved into a synergistic counterpart to experiment, enabling the interpretation of spectral features in terms of molecular motifs and physical mechanisms.^3–7^

Computational spectroscopy generally relies on a quantum mechanical (QM) description of the chemical system. In the gas phase, a variety of well-established approaches have been developed, ranging from derivative-based response methods^2^ to real-time propagation techniques,^8–10^ that can predict spectroscopic observables across different regimes with varying degrees of accuracy and computational demand. However, to realistically describe most chemical and biochemical processes, one must consider the condensed phase, with aqueous solution being the most prominent environment. Here, the unique physical and chemical properties of water, such as its polarity, hydrogen bonding capabilities, and dielectric response, can profoundly influence the spectroscopic signatures of solvated species.^11^ Consequently, gas-phase models often fail unless an explicit treatment of the surrounding medium is considered.

To address this challenge, multiscale models have been developed in which the solute is treated quantum mechanically, and the solvent is represented using classical or semiclassical descriptions.^11–17^ Among these, polarizable embedding schemes stand out for their ability to capture the mutual polarization between solute and solvent.^11,18–23^ Polarizable QM/Molecular Mechanics (MM) embedding techniques belong to the family of “focused models”;^11^ the latter represent a targeted approach in which computational accuracy is concentrated on the portion of the system directly involved in the chemical processes of interest, while the rest is treated at a more affordable level of theory. These models, in particular, allow for an accurate description of local electronic properties (such as electronic transitions, excited states, or chemical reactivity) by applying a refined quantum mechanical treatment to the active region (usually small), while maintaining a realistic representation of the surrounding environment through polarizable embedding.^24–26^ It should be noted that the use of polarizable embedding approaches in the context of ultrafast excited-state dynamics is not trivial and may give rise to an unphysical description of time-resolved processes and signals.^18^ In the present work, however, we target the modeling of static spectral signals, for which the linear-response polarizable embedding adopted here is robust, as already demonstrated in several previous studies.^27–31^ Focused models are especially useful for simulating complex systems, such as solvated systems, where a full quantum mechanical treatment would be computationally prohibitive.

In this work, we employ two polarizable QM/MM models: the QM/Fluctuating Charges (QM/FQ)^24,32^ and QM/Fluctuating Charges and Fluctuating Dipoles (QM/FQFμ) methods,^33,34^ together with their extension based on frozen density embedding (FDE),^35,36^ QM/FDE/FQ­(Fμ).^37,38^ These methods enable the accurate and self-consistent inclusion of environmental effects by treating the solvent as a polarizable medium that dynamically responds to the solute’s quantum mechanical charge distribution.^39^ To demonstrate the versatility and effectiveness of these approaches, we apply them to the modeling of a broad range of spectroscopies—including UV/vis absorption,^27^ vibrational (IR^28,40^ and Raman^29,41,42^ ), electronic circular dichroism (ECD),^37,43,44^ optical rotation (OR),^45–47^ Raman optical activity (ROA),^29,41,48^ and nuclear magnetic resonance (NMR).^38,49^ Although individual methods have previously been employed—each to varying extents—to model specific spectroscopic properties,^11,27,28,37,38,41,50,51^ the present work introduces a comprehensive and unified framework capable of addressing a broad range of observables within a consistent multiscale strategy. By combining polarizable QM/(QM’)/MM methodologies with molecular dynamics simulations in a single protocol, we accurately simulate electronic, vibrational, and chiroptical properties, while also highlighting the differences and specific challenges that arise when the framework is applied to diverse spectral signals. Other strategies for including solvation effects on the calculations of spectroscopic properties have been widely employed in the literature,^52^ ranging from continuum models (PCM/CPCM)^53,54^ to explicit solvent molecular dynamics based on MM nonpolarizable fixed-charge force fields such as TIP3P,^54^ or hybrid cluster-continuum approaches where selected solvent molecules are treated at the QM or MM level while the bulk is eventually described as a dielectric medium.^53–60^ These approaches have proven effective and computationally efficient,^61^ but they generally lack an explicit description of mutual solute–solvent polarization. In contrast, the polarizable QM/FQ­(Fμ) and QM/FDE/FQ­(Fμ) models adopted here explicitly include such effects in a self-consistent manner, offering a balanced compromise between accuracy and computational cost.

To highlight the potentialities and quality of the approaches, they are applied to l-tryptophan (TRP) in aqueous solution. TRP is an essential amino acid featuring a distinctive indole chromophore, which renders it highly responsive to various spectroscopic probes. Its relevance spans biochemical, pharmaceutical, and environmental contexts, and its interactions with water are known to significantly affect its structural and electronic characteristics. Moreover, the wealth of experimental data available for tryptophan makes it an ideal benchmark system for validating multiscale quantum/classical methods. By applying our theoretical protocol across diverse spectroscopies, we establish a unified framework for accurately simulating the spectroscopic behavior of solvated systems, thereby enhancing our understanding of solvation effects and paving the way for applications to larger and more complex biomolecular environments.

## 2 Theoretical Methodology

The spectroscopic signals of tryptophan in aqueous solution are modeled by means of multiscale fully atomistic approaches, based on polarizable QM/FQ­(Fμ) embedding or three-layer polarizable QM/FDE/FQ­(Fμ). In both methods, the solute is treated at the QM level, specifically at the Density Functional Theory (DFT) and its time-dependent (TDDFT) extension. While in QM/FQ­(Fμ) the whole solvent is described at the classical FQ­(Fμ) level,^33^ in QM/FDE/FQFμ the solvent molecules that are closest to the solute are treated at the FDE level,^35^ and the remaining ones at the FQFμ level.^37^ A graphical depiction of the partitioning of the two approaches is provided in Figure 1. The two methods mainly differ in the description of the short-range interactions. In particular, in polarizable QM/FQFμ, the solute–solvent interactions are approximated to electrostatics and polarization effects, under the assumption that nonelectrostatics, such as Pauli repulsion and dispersion, play a minor role, especially for polar solvents,^14^ such as water. Differently, in QM/FDE/FQ­(Fμ), part of the nonelectrostatic interactions, i.e., Pauli repulsion, are taken into account by the FDE layer.^37^

**Figure 1:**
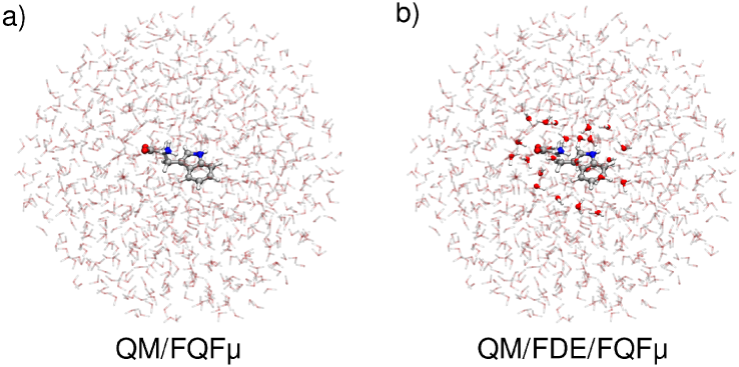
Graphical depiction of QM/FQFμ (a) and QM/FDE/FQFμ (b) partitioning for TRP in aqueous solution.

The energy of the total system can be written as^38,51^

1 $$E=E_{\mathrm{QM}(/\mathrm{FDE})}+E_{\mathrm{FQF}\mu }+E_{\mathrm{QM}(/\mathrm{FDE})/\mathrm{FQF}\mu }^{\mathrm{int}}$$

where *E*_QM(/FDE)_ and *E*_FQFμ_ are the energies of the QM­(/FDE) and FQFμ layers, respectively, whereas $E_{\mathrm{QM}(/\mathrm{FDE})/\mathrm{FQF}\mu }^{\mathrm{int}}$ represents the interaction energy between the two parts. The QM/FQ and QM/FQFμ models account for mutual solute–solvent polarization effects by endowing each classical atom in the environment with charges *q* and dipoles μ that adjust to the QM density.^11,33^ Specifically, in the QM/FQ model, the polarization response of the classical region is described via fluctuating atomic charges determined through the Electronegativity Equalization Principle (EEP).^62,63^ In QM/FQFμ, additional dipoles are introduced to account for anisotropic polarization.^33^ A set of Lagrangian multipliers λ is included in both force fields to ensure the charge neutrality of each solvent molecule.

The interaction energy $E_{\mathrm{QM}(/\mathrm{FDE})/\mathrm{FQF}\mu )}^{\mathrm{int}}$ in eq 1 can then be written as the electrostatic interaction between the solvent multipolar moments and the total electric potential and field generated by the QM­(/FDE) subsystems. By defining ρ_tot_ as the total density of the QM (and FDE) portion, $E_{\mathrm{QM}/\mathrm{FDE}/\mathrm{FQF}\mu }^{\mathrm{int}}$ can be written as

2 $$\begin{array}{ll} E_{\mathrm{QM}(/\mathrm{FDE})/\mathrm{FQF}\mu }^{\mathrm{int}} & =\overset{\mathrm{MM}}{\underset{i}{\sum }}q_{i}V\left[\rho_{\mathrm{tot}}\right]\left(r_{i}\right)-\mu_{i}\cdot E\left[\rho_{\mathrm{tot}}\right]\left(r_{i}\right)\\ & =q_{\lambda }^{†}V\left[\rho_{\mathrm{tot}}\right]-\mu^{†}E\left[\rho_{\mathrm{tot}}\right] \end{array}$$

where *i* runs over the atoms of the MM part. *V*[ρ_tot_]­(**r**_*i*_) and **E**[ρ_tot_]­(**r**_*i*_) are the QM potential and field evaluated at position **r**_*i*_ of the *i*-th atomic MM site.

We can now rewrite eq 1 in terms of the energy functional of FQFμ force field (FF)^33^ and the interaction energy defined in eq 2:

$$\begin{array}{cc} E_{\mathrm{QM}(/\mathrm{FDE})/\mathrm{FQF}\mu }\left[\rho_{\mathrm{tot}},q,\lambda ,\mu \right] & =E_{\mathrm{QM}(/\mathrm{FDE})}\left[\rho_{\mathrm{tot}}\right],+\frac{1}{2}q_{\lambda }^{†}Mq_{\lambda }+q_{\lambda }^{†}C_{Q}+q^{†}V\left[\rho_{\mathrm{tot}}\right],+q^{†}T^{q\mu }\mu +\frac{1}{2}\mu^{†}T^{\mu \mu }\mu -\mu^{†}E\left[\rho_{\mathrm{tot}}\right] \end{array}$$ 3

where, **q**_λ_ is a vector containing FQ charges **q** and a set of Lagrange multipliers **λ**, that fix the total charge *Q* on each FQ molecule. **M** is a matrix collecting the charge–charge interaction kernel (**T** ^*qq*^) and a set of Lagrangian blocks.^64^ **T** ^*qq*^ diagonal elements describe charge self-interaction energy and are expressed in terms of atomic chemical hardnesses η. **C**_*Q*_ is a vector containing atomic electronegativities **χ** and charge constraints **Q**. The FQFμ FF additionally accounts for charge-dipole and dipole–dipole interactions, which are expressed in terms of **T** ^*qμ*^ and **T** ^*μμ*^ interaction kernels.^65^ The diagonal elements of the **T** ^*μμ*^ tensor represent the dipole–dipole self-interaction and are written in terms of the atomic polarizabilities α. Notably, QM­(/FDE)/FQ is obtained by retaining the terms exclusively depending on charges in eq 3.^37^

In accordance with the EEP principle, the polarizable charges (and dipoles) are finally obtained by a constrained minimization of eq 3 with respect to charges (dipoles) and Lagrange multipliers, yielding the following linear system of equations:
4

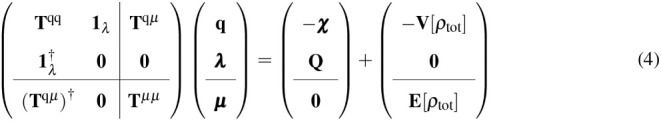

where the second term on the right-hand side describes the mutual polarization between the QM­(/FDE) and FQFμ regions. A functional differentiation of eq 3 with respect to the solute density function (ρ_*I*_ in QM/FDE/FQFμ; ρ in QM/FQFμ) gives the Kohn–Sham (KS) Hamiltonian that defines the Self-Consistent Field (SCF) procedure. In both cases, this results in the addition of the following operator:^24,33^

5 $${Ĥ}_{\mathrm{QM}(/\mathrm{FDE})/\mathrm{FQF}\mu }=\underset{i}{\sum }\frac{q_{i}}{\left|r_{i}-r\right|}-\underset{i}{\sum }\mu_{i}\frac{r_{i}-r}{|r_{i}-r{|}^{3}}$$

where charges and dipoles are obtained by solving eq 4.

The computational description of molecular properties and spectra in (aqueous) solutions can be largely modified by the quality of the modeling of solute–solvent interactions, being particularly crucial the inclusion of solute–solvent polarization effects (which are featured by QM/FQFμ).^11,21,22,51^ To further extend QM­(/FDE)/FQFμ to response properties of solvated systems, the modification of the ground state (GS) molecular orbitals (MOs), which results from the SCF procedure (see eq 5), is not sufficient. A generic response property can be computed by solving the proper response equations,^66,67^ which must be modified in order to account for mutual polarization effects resulting from the perturbation of the QM density. In a DFT framework, this means that the embedding method has to be extended to electric and magnetic Coupled-Perturbed KS (CPKS) equations,^66^ accounting for the adjustment of the polarization sources to the perturbed density, and specific terms due to the usage of Gauge—including atomic orbitals (GIAOs) in magnetic response.^68^ Absorption spectra are instead computed by including specific polarization terms into Casida’s equations,^69^ in order to account for the dynamic response (linear regime) of polarization sources (we refer the reader to ref 27 for a detailed discussion on modeling absorption spectroscopy using polarizable QM/MM methods). For vibrational response, nuclear perturbation must also be considered, extending the method to the analytical evaluation of energy first derivatives (gradients) using the machinery of quantum chemistry.^70^ Second and higher order derivatives can be computed by using analytical or numerical differentiation, opening the way to the simulation of IR,^28,40^ Raman,^29,41^ and Raman Optical Activity spectra.^29,41^

## 3 Results and Discussion

### 3.1 Computational Protocol

The application of QM/(FDE)/FQ­(Fμ) to the calculation of molecular properties and spectral signals of aqueous TRP requires its coupling to a specific multistep protocol, which has been proposed and refined in recent years.^11,27^ Similar strategies, based on the combination of MD sampling with quantum chemical calculations of several spectroscopic properties in solution, have also been employed in previous works.^71–74^ The protocol consists of the following steps:

(1) *Definition of the system:* TRP (in its zwitterionic form) is treated at the QM level, whereas the surrounding water environnment is treated classically, by using FQ and FQFμ.
(2) *Conformational and configurational sampling:* TRP-water configurational space is explored through classical molecular dynamics (MD) simulations performed using the GROMACS package^75^ and conducted on the nanosecond time scale. Both intramolecular and intermolecular interactions are treated using the General Amber Force Field (GAFF2),^76^ while the standard TIP3P force field^77^ is used to describe the solvent. TRP initial geometry is chosen to match the optimized structure at the B3LYP/aug-cc-pVDZ level of theory, and then solvated by preparing a cubic simulation box with a side length of 5.9 Å, containing 6864 water molecules. CM5 charges are employed.^78^
  To avoid atomic overlap and unrealistic interactions between molecules, the system’s energy is minimized. Subsequently, before starting the production step, two equilibration stages are performed. First, a 1 ns NVT equilibration stage is run, maintaining the temperature constant at 298.15 K using a velocity-rescaling thermostat^79^ with a coupling constant of 0.1 ps and an integration time step of 2.0 fs. Then, a 2 ns NPT equilibration stage is carried out, maintaining the pressure at 1.0 bar using the Parrinello–Rahman barostat^80^ with a coupling constant of 2.0 ps and an integration time step of 2.0 fs. Finally, an NPT production stage is run for 50 ns with an integration time step of 2.0 fs, using the LINCS algorithm^81^ to maintain all bond lengths constrained, allowing for stable and efficient simulations. The particle-mesh Ewald (PME)^82^ method is used to handle electrostatic interactions, with a grid spacing of 0.16 nm and a spline interpolation of order 4. Short-range Coulomb and van der Waals interactions are truncated using the Verlet cutoff scheme,^83^ with a cutoff distance of 1.2 nm.
(3) *Extraction of structures*: From the production stage of the MD simulation, several uncorrelated snapshots are extracted every 10 ps.^84–91^ Then, solute–centered spheres (droplets) of radius equal to 18 Å are cut. This dimension is large enough to retain solute–solvent interactions in a physically consistent way.
(4) *QM/FQ*(*F*μ) *calculations of the target molecular properties/spectra*: Calculations of the target response/spectral properties are performed on a variable number of spherical droplets obtained at the previous step. The number of droplets is chosen to ensure numerical convergence of the results. UV and ECD spectra are simulated by processing respectively 300 and 950 snapshots at the TD-CAMY-B3LYP/TZ2P level of theory (see Figures S3–S6). The choice of this functional is consistent with previous studies demonstrating the reliability of range-separated hybrids for these properties, including applications to l-tryptophan.^92,93^ NMR, IR, Raman, ROA, and OR calculations are performed at the B3LYP/TZ2P level of theory, in line with several previous works where hybrid functionals have been successfully applied to vibrational and magnetic properties of l-tryptophan.^94–96^ While 200 snapshots are sufficient for NMR, IR, Raman, and ROA, a significantly larger number—4000 snapshots—is necessary to achieve convergence in OR (see Tables S1,S2 ,Figures S10, S11 and S13–S18).
  FQ and FQFμ layers are modeled by exploiting the parametrization reported in refs 33 and 97.
(5) *Data analysis and extraction of final properties/spectra:* For UV and ECD, the final spectra are obtained by applying a Gaussian convolution with a Full Width at Half Maximum (fwhm) of around 0.5 eV to averaging data. In the case of ^1^H NMR, the full spectrum is computed by including nuclear spin–spin couplings under an external magnetic field of 600 MHz. To generate the final spectral profile, the resulting chemical shift values are convoluted using a Lorentzian function with a fwhm of 0.002 eV. In contrast, for ^13^C, ^15^N, and ^17^O NMR, spin–spin couplings are not considered. Accordingly, only average values obtained from the QM/FQ and QM/FQFμ calculations are reported. Specifically, chemical shifts are provided for ^13^C, whereas nuclear shielding constants are reported for ^15^N and ^17^O. Chemical shifts are obtained from computed shielding constants using tetramethylsilane (TMS) as reference, with σ_ref_ = 31.7 ppm for ^1^H and σ_ref_ = 182.852 ppm for ^13^C, as employed in Amsterdam Modeling Suite (AMS).^98^ For IR, Raman, and ROA spectra, stick spectra obtained from individual snapshots are averaged and convoluted using a Lorentzian function with a fwhm of 10 cm^–1^. A Raman excitation energy of 634 nm is used to simulate the spectra. In addition to QM/FQ and QM/FQFμ, for NMR, a QM/FDE/FQFμ approach is employed. The FDE protocol consisted of three sequential steps: (i) a full-system QM/MM calculation with the solute and all solvent molecules, (ii) a QM/FQFμ single-point calculation on the water molecules within the FDE layer (treated at QM level), embedded in the remaining solvent molecules (described at FQFμ level), to generate a frozen electron density (ρ_2_), and (iii) a final QM/FDE/FQFμ calculation where this frozen density is used to represent the inner solvent shell, while the remaining solvent is treated as a polarizable MM region. In step (iii), to calculate the FDE terms, the PW91K86 functional^99^ is used to approximate the kinetic energy, while the nonadditive exchange-correlation terms are treated using the PBE functional.^36^ The selection of PW91K is based on previous studies that demonstrated its effectiveness in simulating ground-state properties and NMR spectra with FDE.^38,100–102^ QM/FDE/FQFμ calculations are performed using an FDE radius of 3Å and incorporating freeze-and-thaw (FT) cycles.^103^
  As regards OR calculations, a single value corresponding to the sodium D-line energy (589.3 nm) is computed for each snapshot across both QM/FQ and QM/FQFμ methods, highlighting the frame to frame variation of that property.
  All QM/FQ­(Fμ) and QM/FDE/FQ­(Fμ) calculations are performed with the AMS.^104,105^

### 3.2 Conformational Analysis and Hydration Patterns

TRP is a flexible molecule, and its conformation is expected to be affected by the presence of the aqueous environment, which can also interact with TRP by forming hydrogen bonding (HB).

Two main factors ensure a high-quality conformational sampling for the TRP aqueous system, i.e. (i) the sampling of the solute conformation in terms of the internal dihedral angles within the molecule that originate from intramolecular interactions (such as weak noncovalent interactions, HB, ...), and (ii) the sampling of the solvent configurations and the distribution and orientation of solvent molecules around the solute.^11^

The MD trajectory is initially analyzed using clustering analysis^106^ of GROMACS to identify the main configurations of TRP, employing a root-mean-square deviation (RMSD) threshold of 0.11 nm. Four primary conformers are identified, shown in Figure S1, with relative populations of 66%, 18%, 7%, and 4%, respectively. TRP is characterized by three key dihedral angles: δ_1_, δ_2_, and δ_3_—the definitions and specific values of the representative structures are detailed in Figure 2b and S1. The dihedral distribution functions (DDFs) for δ_1_, δ_2_, and δ_3_ are presented in Figure S2. δ_1_, the dihedral angle connecting the aromatic ring to the main chain, shows a dominant peak centered at approximately −60° and a secondary peak centered at about 100°, which indicates some rotational flexibility around this bond. Similarly, δ_2_, which links the main chain to the amino group, shows a dominant peak near −60°, along with minor peaks at around 60° and ± 180°. δ_3_, connecting the amino group to the carboxyl group, shows broad peaks centered at −60° and 130°, reflecting significant rotational flexibility. The δ_1_ and δ_2_ values obtained from the main clusters, identified through the clustering analysis, show good agreement with the representative conformers reported in the literature (see Figure 2a).^107^ As shown in the figure, both our clusters and those from the literature fall within the high-density regions of the dihedral angle distribution, indicating that they represent the most populated conformational basins sampled during the simulation. No clusters fall within the low-density regions, which is expected since only the most representative structures are selected. The third dihedral angle, δ_3_, is not included in the 2D analysis due to its high flexibility, as it spans nearly the entire angular range (see Figure S2). Overall, these results support the consistency between different sampling and analysis strategies and confirm the reliability of our conformational model.

**Figure 2:**
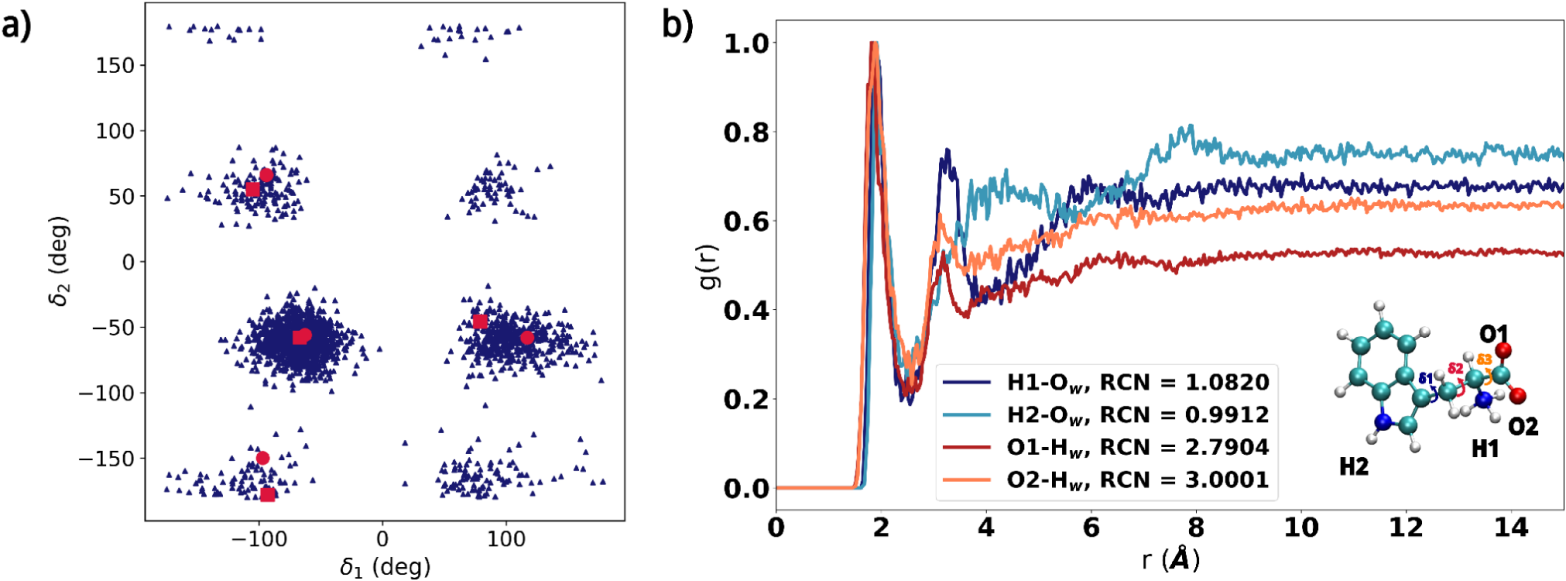
(a) Left: MD conformational analysis (blue triangles) for TRP zwitterionic form in aqueous solution as a function of δ_1_ and δ_2_. The conformations of the 4 main clusters obtained through clustering analysis are also shown as red circles, while the red squares represent the dihedrals of the lowest energy conformers reported in the literature.^107^ (b) Right: Radial distribution functions (RDFs) between TRP and water molecules, with the corresponding running coordination numbers (RCNs). The definition of δ_1_, δ_2_ and δ_3_ is also reported.

To analyze solute–solvent HB interactions, radial distribution functions *g*(*r*) (RDFs) and running coordination numbers (RCNs) are extracted from the MD trajectory (see Figure 2b). Around oxygen atoms of the carboxyl group (O1 and O2), there is a first solvation shell at about 1.82 Å (O1) and 1.88 Å (O2), respectively, with corresponding RCNs of 2.79 and 3.00. This indicates that the carboxyl group forms, on average, HBs with three water molecules. In the case of the hydrogen atoms of the amino group (H1) and the pyrrolic group (H2), the first solvation shell is placed at approximately 1.85 Å for H1 and 1.91 Å for H2, with RCNs of 1.08 and 0.99, reflecting the formation of an HB with a single water molecule. A similar analysis can be performed to investigate potential intramolecular HBs between the amino and the carboxyl groups. No significant peaks are detected, thus indicating the absence of persistent intramolecular HBs within TRP, and also suggesting that the solute primarily forms HBs with the solvent molecules.

### 3.3 UV and ECD Spectra

This section presents computed QM/FQ (Fμ) UV and ECD spectra of TRP in aqueous solution. Figure 3 displays the calculated stick spectra, representing the raw excitation energies and corresponding oscillator strengths obtained from individual snapshots extracted from the MD trajectory. Each snapshot produces a distinct spectral signal as a result of the varying configurations of the solute and different distributions of the solvent around it, effectively capturing solvent-induced inhomogeneous band broadening without the need for ad-hoc corrections.

**Figure 3:**
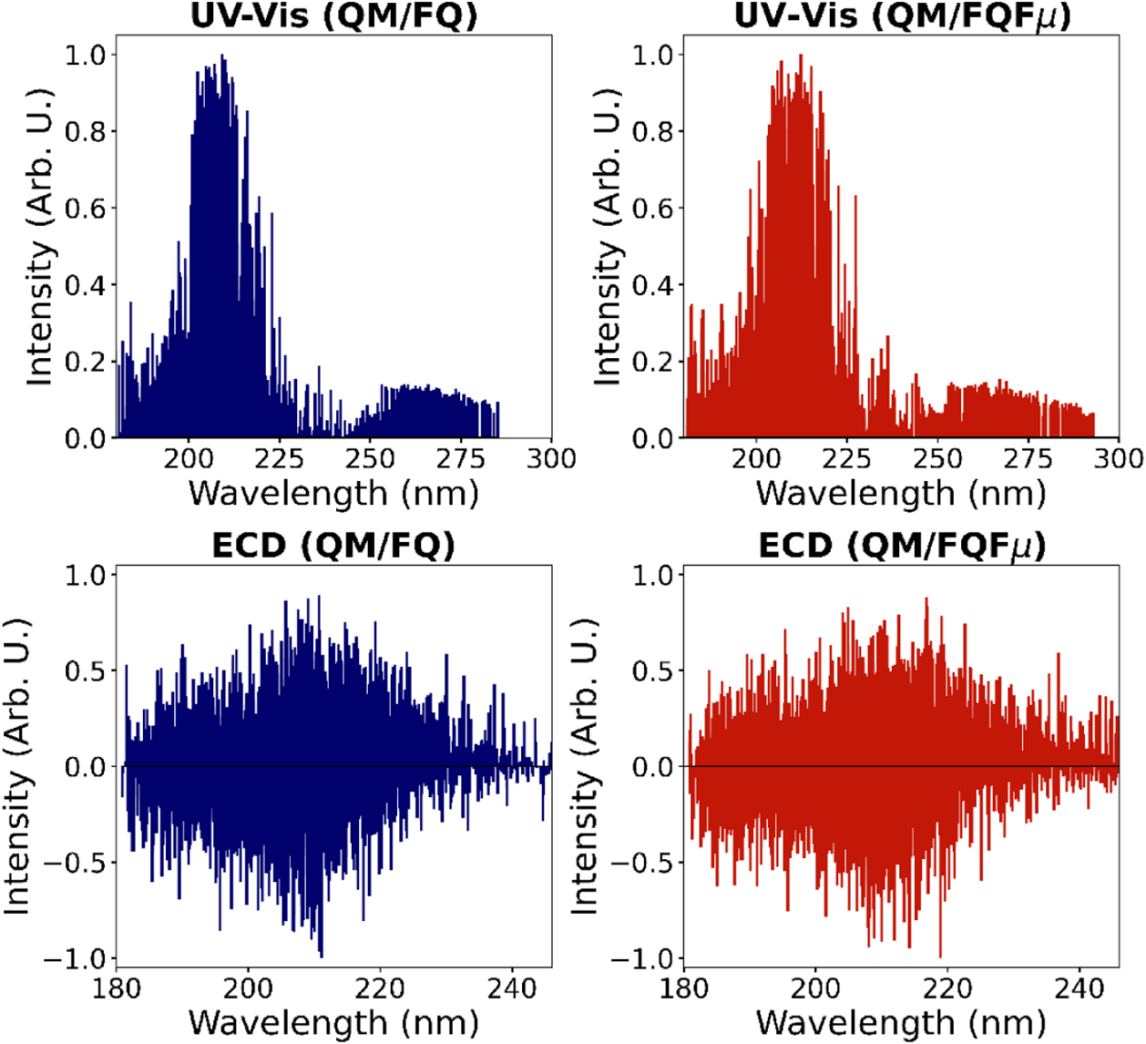
UV and ECD QM/FQ (left) and QM/FQFμ (right) stick spectra of l-tryptophan (TRP) in aqueous solution, derived from 300 (for UV) and 950 (for ECD) structures extracted from the MD simulation.

QM/FQ (Fμ) UV and ECD spectra are shown in Figure 4. The results of the study of the convergence of computed spectra with respect to the number of snapshots are reported in Figures S3–S6 in the SI. Convergence of averaged values is achieved employing 200 snapshots for UV and 900 for ECD, at both QM/FQ and QM/FQFμ level. The larger number of snapshots which are required to converge ECD spectra highlights the complex interplay of computed positive and negative rotational strengths (see Figure 3), and aligns with previous studies of some of us.^44,51^

**Figure 4:**
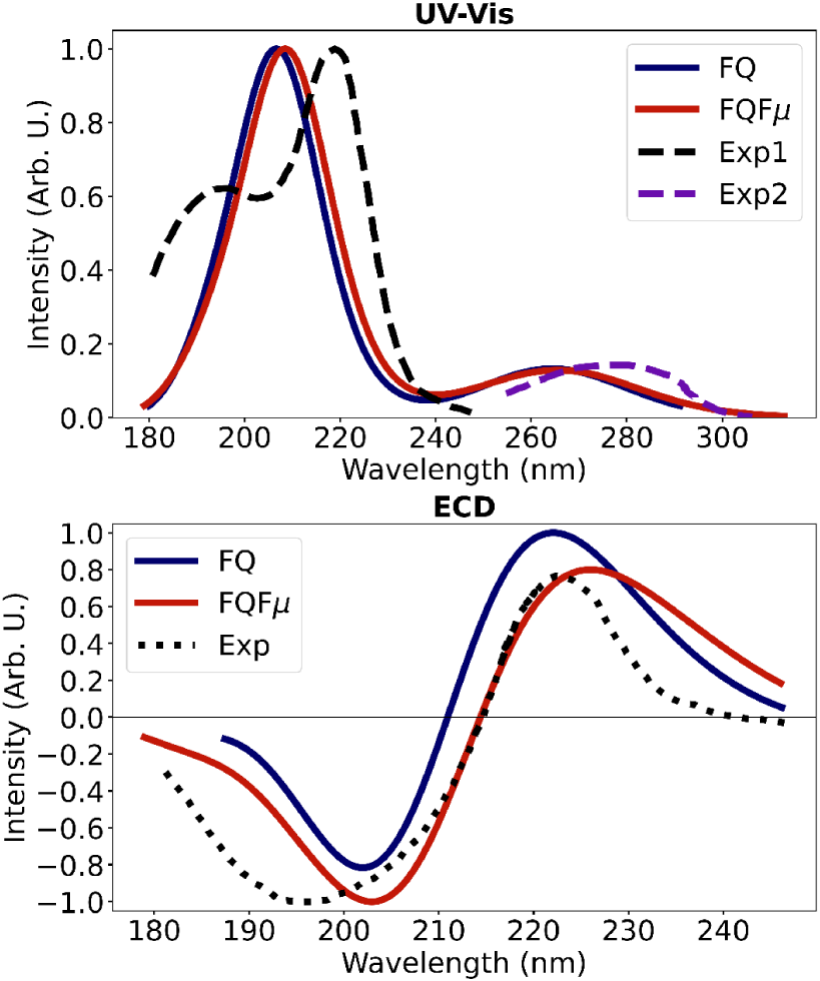
QM/FQ and QM/FQFμ UV (top) and ECD (bottom) spectra of l-tryptophan (TRP) in aqueous solution, derived from 300 (for UV) and 950 (for ECD) structures extracted from the MD simulation and computed at the CAMY-B3LYP/TZ2P level of theory. A Gaussian convolution having a fwhm of 0.5 eV is exploited to draw the spectra. Computed data are compared with experimental UV spectra^108,109^ and experimental ECD spectrum.^108^

The averaged QM/FQ (Fμ) UV spectra (see Figure 4) exhibit an intense high-energy absorption peak accompanied by a weaker peak at lower energy. The corresponding ECD spectra, also depicted in Figure 4, display a distinctive (−, +) sign pattern for both solvent models. Canonical molecular orbital (CMO) decomposition, based on natural bonding orbital (NBO) analysis,^110,111^ reveals that the lower-energy peak originates from the HOMO → LUMO π → π* electronic transition. CMOs involve orbitals localized in the aromatic ring of TRP, as shown in Figure S7. Notice that the NBO analysis is performed on the representative structure of the main conformer of TRP, obtained through the GROMACS cluster analysis, as described in Section 3.2. Solvent effects are implicitly considered by employing the COSMO continuum solvent model.^112,113^

Only minor differences between QM/FQ and QM/FQFμ UV and ECD spectra are observed. The inclusion of fluctuating dipoles in QM/FQFμ leads to a slight redshift in peak positions, while the shape of the bands and the relative intensities remain largely unchanged. In UV spectra, the main peak appears at 206 nm for QM/FQ and 208 nm for QM/FQFμ, with a weaker band at 264–265 nm. Both ECD spectra display the characteristic (−, +) pattern, with bands at 201/223 nm for QM/FQ and 203/226 nm for QM/FQFμ.

Figure 4 reports also experimental UV and ECD spectra, enabling a direct comparison between theoretical predictions and experimental observations. The experimental UV data for l-tryptophan in aqueous solution are taken from two distinct measurements covering different spectral regions. One experiment reports the high-energy transitions,^108^ which give rise to two bands centered at 190 and 220 nm, with the latter being the more intense. The other experiment focuses on the near-UV region,^109^ showing a broad band centered at about 278 nm. As shown in Figure 4, the simulations predict only one band in the high-energy region, while the experimental spectrum resolves two contributions at 190 and 220 nm, and they slightly overestimate the position of the 278 nm band, possibly due to intrinsic limitations of DFT. Overall, the computed spectra reproduce pretty well the positions of the experimental peaks and the observed band broadening. The ECD experimental spectrum^108^ shows a negative peak at around 200 nm, followed by a positive peak near 220 nm. Both computed spectra accurately reproduce the key features of the experimental spectrum, especially the sign pattern (−, +) that is the most important characteristic of chiral spectroscopies. Furthermore, the calculations yield excellent agreement in terms of peak positions and band broadening for both QM/FQ and QM/FQFμ methods, while the agreement in relative intensities is observed only with the QM/FQFμ approach. These results improve upon those previously reported in the literature using PCM TDDFT/CAM-B3LYP or TDDFT/B3LYP with the aug-cc-pVDZ basis set, where the final spectrum was obtained by Boltzmann averaging over the nine most stable conformers optimized by the authors.^93^ In particular, our protocol not only reproduces the experimental alternation of signs (−, +), as already achieved in earlier studies, but also provides a more accurate description of both peak positions and band broadening.

### 3.4 NMR Spectra

Computed QM/FQ (Fμ) chemical shifts for TRP hydrogen and carbon nuclei, as well as shielding constants for nitrogen and oxygen, are reported in Table 1 and Figure 5 for atom labeling. Tabulated values are obtained by averaging computed values for 200 snapshots, to ensure convergence (see Tables S1 and S2).

**Figure 5:**
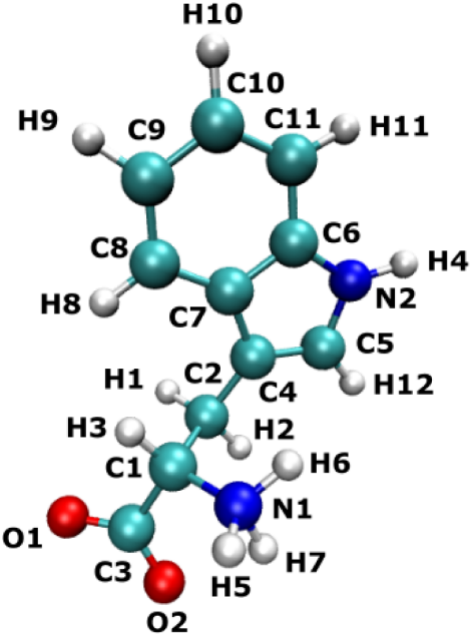
Atom labeling scheme of TRP used for chemical shift and shielding assignment.

**Table 1:** QM/FQ and QM/FQF*μ* Computed Chemical Shifts (ppm)­(for Hydrogen and Carbon) and Magnetic Shielding Constants (for Nitrogen and Oxygen)^a^,^b^

Atom	QM/FQ	QM/FQFμ
**Hydrogens**		
H1	3.7336	3.6629
H2	3.4384	3.4743
H3	4.3763	4.3996
H4	10.0063	10.4016
H5	6.0134	6.2879
H6	5.9277	6.1879
H7	6.3728	6.7483
H8	8.2038	8.2446
H9	7.6214	7.6119
H10	7.7782	7.7860
H11	8.0512	8.1204
H12	7.9716	8.0644
**Carbons**		
C1	63.9518	63.5953
C2	30.4370	30.4645
C3	176.2754	178.5201
C4	117.7629	117.2523
C5	135.2466	136.4871
C6	143.8707	143.8123
C7	132.6885	132.6688
C8	124.3865	124.5879
C9	127.0266	126.6992
C10	129.4505	129.1790
C11	121.6423	122.0687
**Nitrogens**		
N1	210.4982	211.7675
N2	104.0095	101.8629
**Oxygens**		
O1	35.1612	42.1765
O2	48.8983	55.5504

a Values are Obtained by Averaging Over 200 Snapshots of Solvated TRP.

b QM Level of Theory: B3LYP/TZ2P.

For ^1^H nuclei, the differences between QM/FQ and QM/FQFμ are small, as evidenced by the average absolute deviations reported in Table S3. The maximum intermodel deviation for hydrogen nuclei is 0.4 ppm, observed for the N–H proton of the pyrrole ring H4 (see Figure 5).

In comparison to QM/FQ, the inclusion of dipoles in the solvent description alters the electron density around the nuclei, leading to upfield or downfield shifts. A noticeable deshielding of 0.3–0.4 ppm is observed for nuclei that strongly interact with the aqueous environment, such as H4, H5, H6, and H7, as a consequence of the enhanced solute–solvent electrostatic interaction described by QM/FQFμ compared to QM/FQ.

Consistently with the trends observed for ^1^H, also in the case of ^13^C nuclei the differences among the QM/FQ and QM/FQFμ models are generally low and range between 0.02 and 1.0 ppm for most nuclei, with the exception of carboxylic carbon (C3) and C5 (see Table S3 ).

The averaged data for oxygen and nitrogen nuclei are also presented in Table 1. Due to their strong interactions with the surrounding medium, oxygen nuclei exhibit high sensitivity to the choice of solvent model, with large variations across the different approaches, and a maximum difference of around 7 ppm observed between QM/FQ and QM/FQFμ. Conversely, nitrogen nuclei show a lower sensitivity, following a trend similar to hydrogen and carbon, with a maximum shielding variation of approximately 2 ppm for the pyrrolic nitrogen N2, as observed when comparing QM/FQ and QM/FQFμ.

NMR chemical shifts and shielding constants are local magnetic response properties, strongly influenced by the electronic environment around the nucleus. They are particularly sensitive to short-range effects such as hydrogen-bond directionality and local electronic polarization. To better account for these interactions—especially those involving the first solvation shell—we employed a hybrid QM/FDE/FQFμ model. In this scheme, solvent molecules within a certain radius of the solute are treated quantum mechanically using the FDE approach, while the remaining environment is described by a polarizable molecular mechanics layer (FQFμ). This enables a more accurate representation of the local electronic environment without requiring a full QM treatment of the entire solvent.^38^ To assess the reliability and cost-efficiency of this setup, a series of tests is performed on 10 frames by systematically varying key parameters. Specifically, we evaluated the effect of the FDE radius (3, 4, and 5 Å), the use of a mixed basis set in step (ii), and the inclusion of FT cycles in step (iii) (see Section 3.1 for the steps). The influence of the FDE radius and the use of mixed basis sets is first examined (see Figure S8), revealing that the mixed basis has a negligible effect on the computed chemical shifts. Subsequently, the effect of the FDE radius was further investigated in combination with FT cycles (Figure S9 ), highlighting a non-negligible sensitivity for nuclei such as H4–H7, O1, O2, and C3. Among the tested setups, the configuration with a 3 Å FDE radius with FT cycles showed good agreement with the reference calculation (5 Å with FT), while keeping the computational cost low. These settings are therefore adopted for the NMR property calculations.

Similarly to QM/FQ and QM/FQFμ computed NMR spectra, also for QM/FDE/FQFμ model, the final chemical shifts and shieldings values are obtained by averaging over 200 snapshots, which ensures the convergence of the computed values (see Table S4). As shown in Table S5, the inclusion of the FDE layer has little impact on calculated chemical shifts of the hydrogen and carbon nuclei, with the exception of H4–H7 and C3, which show differences of approximately 0.5–0.6 and 4 ppm, respectively, between QM/FQFμ and QM/FDE/FQFμ (see Table S6). As expected, a much more pronounced effect is observed for the oxygen nuclei, with deviations up to 16 ppm compared to QM/FQFμ values. Moving to ^13^C, the subsequent inclusion of the FDE layer induces the same opposing trend observed for ^1^H, resulting in a pronounced shielding effect of up to 4 ppm for C3.

To end the discussion, computed data are compared with available experimental values (see Figure 6). The experimental chemical shifts for hydrogen and carbon nuclei of l-tryptophan in aqueous solution are also reported in Table S13.^114,115^ To maintain consistency with experimental conditions, hydrogen atoms bound to nitrogen (H4, H5, H6, and H7) are excluded from the coupled spectra. For ^1^H nuclei, NMR coupled spectra are simulated assuming a magnetic field strength corresponding to a proton resonance frequency of 600 MHz. The J-coupling constants are computed for all models, as reported in Tables S7–S9.

**Figure 6:**
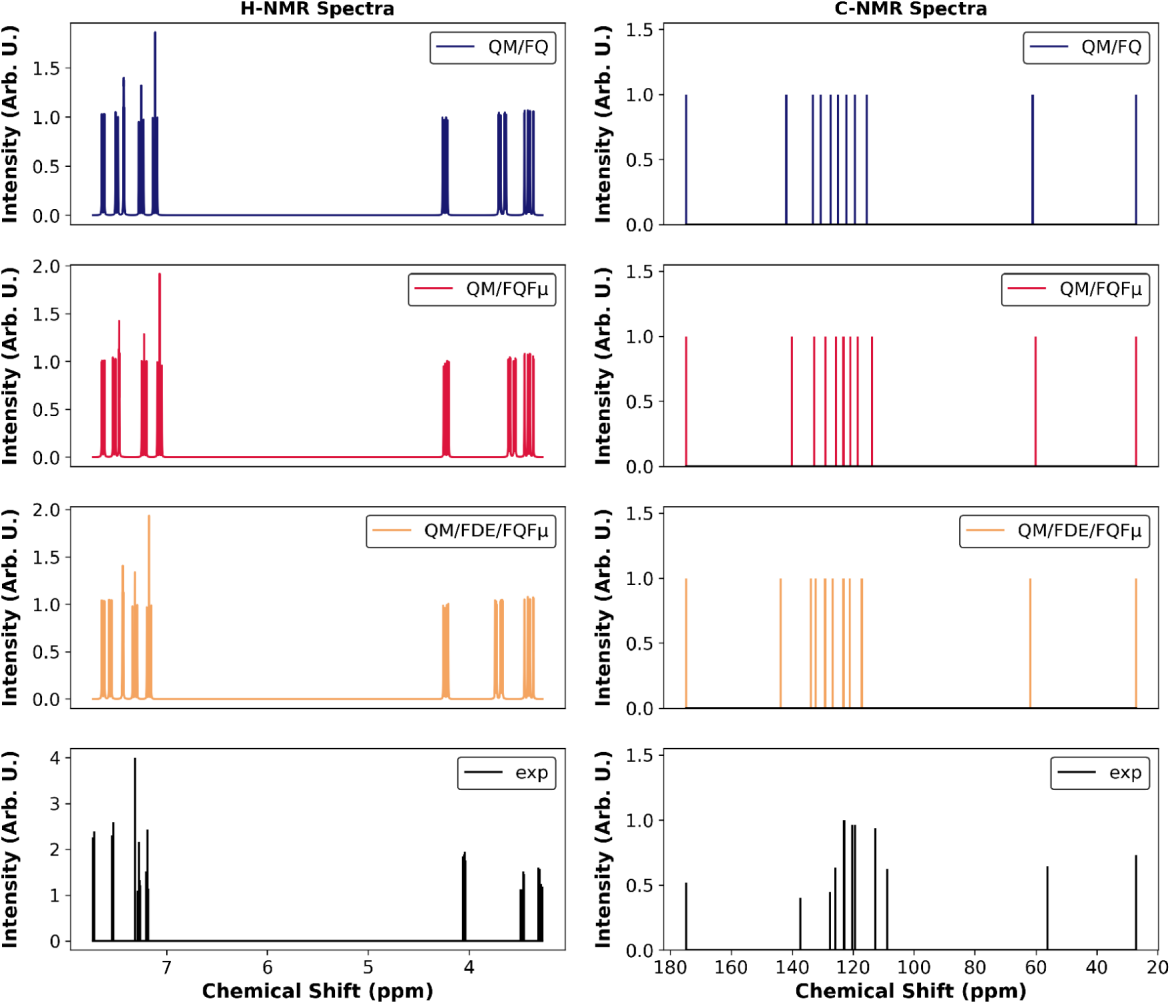
Computed coupled ^1^H NMR and uncoupled ^13^C NMR spectra of l-tryptophan (TRP) in aqueous solution. QM/FQ, QM/FQFμ and QM/FDE/FQFμ approaches are compared. QM level: B3LYP/TZ2P. Values are averaged over 200 snapshots. Chemical shifts are obtained from computed shielding constants using tetramethylsilane (TMS) as reference, with σ_ref_ = 31.7 ppm for ^1^H and σ_ref_ = 182.852 ppm for ^13^C, as employed in AMS.^98^ Experimental NMR spectra are also shown for comparison.^114,115^

Hydrogen atoms H8–H9–H10-H11 of the benzene ring show ortho J-couplings (J_ortho_) of approximately 7–8 Hz. Additionally, long-range couplings, such as ^5^J (e.g., H8–H4), are observed for the indole ring, although J values are lower than 1 Hz, due to the extensive resonance delocalization that facilitates spin–spin interactions over greater distances.

For the aliphatic chain, the highest coupling constants correspond to geminal couplings (^2^J) (H1–H2, H5–H6, H6–H7, H5–H7), ranging from 17 to 19 Hz, while vicinal couplings (^3^J) (H1–H3, H2–H3, H3–H5, H3–H6, H3–H7) fall within the 4–12 Hz range.

J-coupling constants computed with the three models exhibit minimal differences, as shown in Tables S10–S12, with a maximum difference of 0.4 Hz observed for the H3–H7 coupling moving from QM/FQFμ to QM/FDE/FQFμ.

Computed ^1^H NMR spectra reveal two distinct chemical shift regions. Hydrogen atoms belonging to the indole moiety (H8–H12) resonate above 7 ppm, while aliphatic hydrogens (H1–H3) appear below 4 ppm. All computational models successfully reproduce the experimental ^1^H NMR spectrum, with a good agreement in both chemical shifts and relative peak intensities. Although experimental J-coupling constants are not available for direct comparison, computed spectra capture the expected coupling patterns.

The computed decoupled ^13^C NMR spectrum, shown in Figure 6, displays aromatic carbon signals between 110 and 150 ppm. Carboxylic carbon (C3), which is highly deshielded, resonates around 180 ppm, consistent with its electron-withdrawing environment. The two aliphatic carbons (C1 and C2) are found at approximately 30 and 60 ppm, respectively, reflecting their different chemical environments adjacent to the indole ring and the amino group. Theoretical models accurately reproduce the experimental peak distribution, particularly within the aliphatic region (30–60 ppm), with deviations generally within a few ppm.

Because of the absence of unambiguous experimental assignments for the aromatic carbon signals, a direct comparison with the computed spectra is not possible. Nevertheless, the spectra are qualitatively in good agreement, again supporting the validity of our computational approach for both hydrogen and carbon NMR spectral predictions.

### 3.5 Optical Rotation

To complement the analysis of ECD and further investigate the chiroptical properties of l-tryptophan in solution, OR is computed. Simulations are performed using both the QM/FQ and QM/FQFμ models at the B3LYP/TZ2P level of theory. A total of 4000 frames extracted from the MD trajectory are used to ensure statistical convergence (see Figures S10 and S11). The specific incident wavelength used in the calculations corresponds to the sodium D line (589.3 nm), which is commonly adopted for direct comparison with experimental measurements. To gain a deeper understanding of the frame-by-frame fluctuations in OR, stick spectra are generated by reporting the OR values for each of the 4000 snapshots extracted from the MD trajectory. The results are depicted in Figure 7 for both QM/FQ and QM/FQFμ calculations. The large fluctuations in both the sign and intensity of the OR values highlight how strongly this property depends on the specific mutual arrangement of the solute and surrounding water molecules. This behavior is typical of OR, which is highly sensitive to small structural and environmental variations.^41,46^ Despite the intrinsic complexity of OR, both QM/FQ and QM/FQFμ successfully reproduce the experimentally observed negative sign of the OR, measured as −31.5 degree/(dm·g/cm^3^) at 23 °C.^116^ The computed values are −64 degree/(dm·g/cm^3^) with QM/FQ and −71 degree/(dm·g/cm^3^) with QM/FQFμ, reflecting a consistent qualitative agreement with experiment.

**Figure 7:**
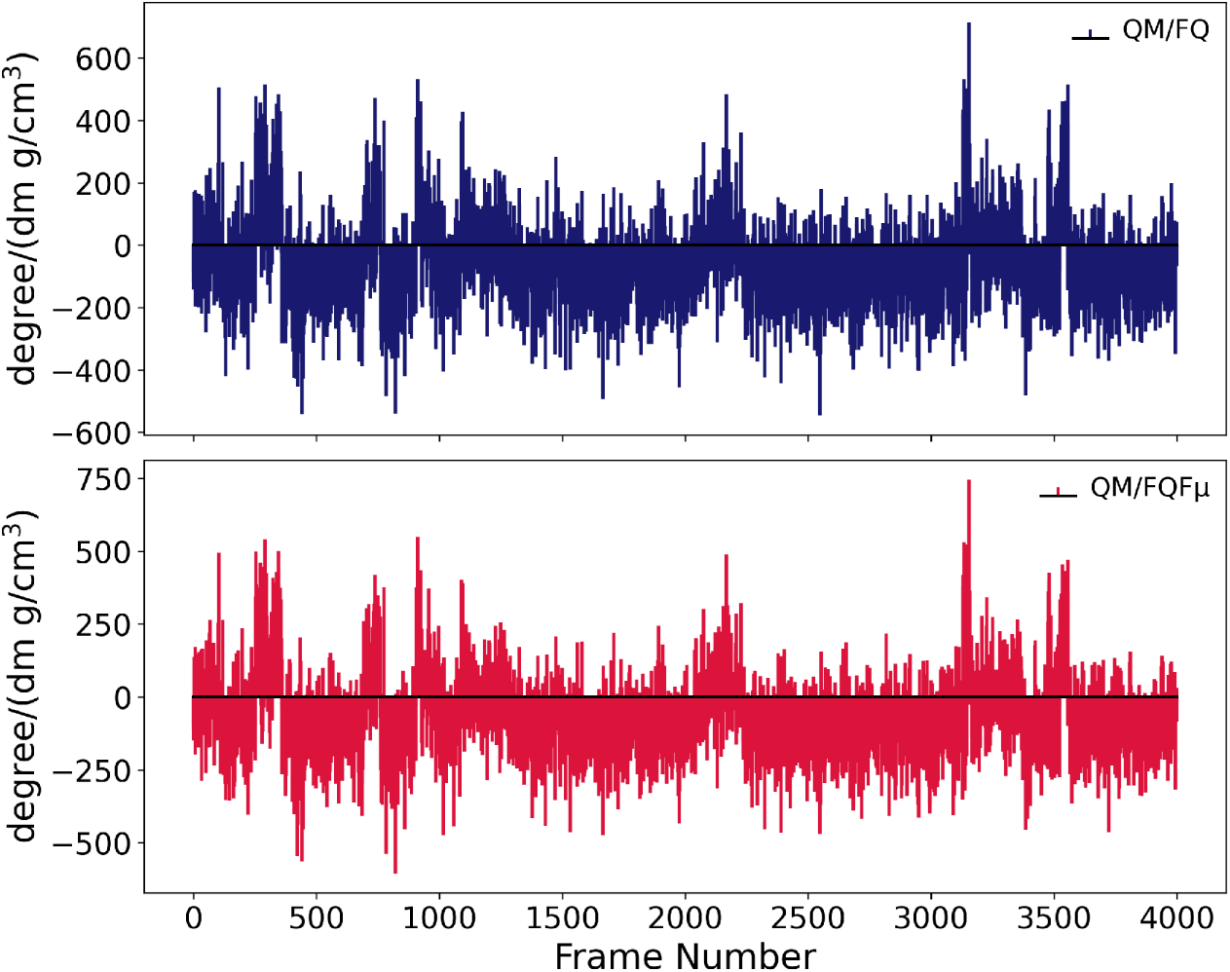
Computed QM/FQ and QM/FQFμ optical rotation at the sodium D-line (589.3 nm) of l-tryptophan in aqueous solution for each of the 4000 frames extracted from the molecular dynamics trajectory. Each stick represents the OR of a single solute–solvent configuration. QM level of theory: B3LYP/TZ2P.

### 3.6 Vibrational Spectra

We now turn our attention to vibrational spectra, which involve a perturbation with respect to nuclear displacements. To stay within the ″focused″ approach that we have discussed in the Introduction, we employ the so-called Partial Hessian Vibrational Analysis (PHVA),^117–119^ which has been used in numerous studies^21,28,48^ and offers a reliable and computationally efficient strategy for vibrational analysis in complex systems.

Within the PHVA, vibrational spectra are computed for the QM active region of the system, in the present case TRP. The influence of the surrounding environment is still accounted for, but only in terms of its effect on the vibrational properties of the active site. This means that not all of the system’s nuclei are included in the vibrational analysis: only the atoms belonging to the active region are used to construct the Hessian matrix, which in turn determines the vibrational modes. Vibrational analysis within the double-harmonic approximation must be performed on energy minima. For this reason, a partial optimization of the various snapshots extracted from the MD is performed, allowing only the QM degrees of freedom to relax and then keeping the solvent frozen. In this way, the sampling of the solvent configuration around the solute achieved in the MD is preserved. It is worth noting that alternative MD-based approaches extract vibrational spectra from time-correlation functions of dipole moments or polarizabilities computed along trajectories.^120–123^ These methods naturally include dynamic effects as well as explicit solute–solvent couplings, however they fully rely on a specific choice of the classical force field. The PHVA offers a harmonic but efficient description of solute vibrations at the full quantum-mechanical level.

The computed stick spectra for IR, Raman, and ROA, obtained with the QM/FQ and QM/FQFμ approaches, are shown respectively in Figures 8 and S12. The results refer to calculations on 200 snapshots, a number that is appropriate to reach convergence on averaged spectra (see Figures S13–S18). For IR and Raman, stick spectra closely reflect the final spectral shapes, while the ROA stick spectrum is more complex to interpret because of the coexistence of positive and negative signals within the same spectral regions, which prevents immediate prediction of the final spectral profile.

**Figure 8:**
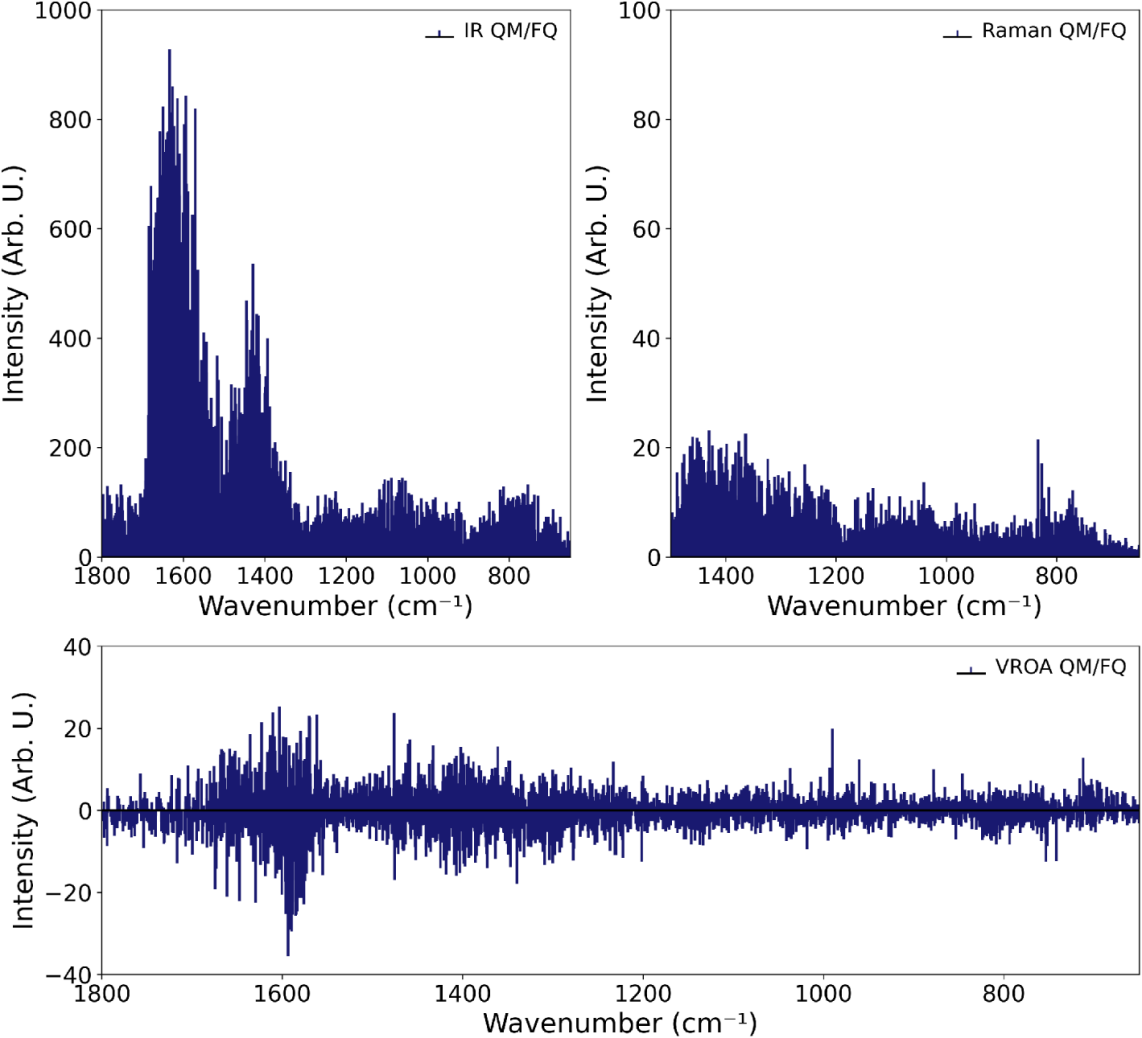
IR, Raman, and ROA stick spectra of l-tryptophan (TRP) in aqueous solution computed using the QM/FQ approach at the B3LYP/TZ2P level of theory. Spectra correspond to normal-mode analyses performed on 200 individual snapshots extracted from the molecular dynamics simulation. IR and Raman spectra are shown in the top panel, and the ROA spectrum in the bottom panel.

The averaged computed IR spectrum (Figure 9) shows three dominant bands centered around 1600 cm^–1^, 1420 cm^–1^, and 780 cm^–1^. The first, and most intense band, is assigned to the asymmetric stretching of the carboxylate group. This mode is coupled with the symmetric deformation of the ${\mathrm{NH}}_{3}^{+}$ group, the out-of-plane bending of the pyrrolic N–H, CH_2_ scissoring, and stretching of conjugated C–C and C–N bonds within the aromatic moiety. The normal modes corresponding to the most prominent vibrational bands are analyzed for the most representative MD frame in Figure S19. The second band, at approximately 1420 cm^–1^, is mainly attributed to the symmetric stretching of the carboxylate group, with additional contributions from aromatic stretching and in-plane C–H bending. The third band at 780 cm^–1^, involves CO_2_ scissoring, out-of-plane bending of aromatic C–H bonds, and rocking motions of the aliphatic CH_2_ and ${\mathrm{NH}}_{3}^{+}$ groups.

**Figure 9:**
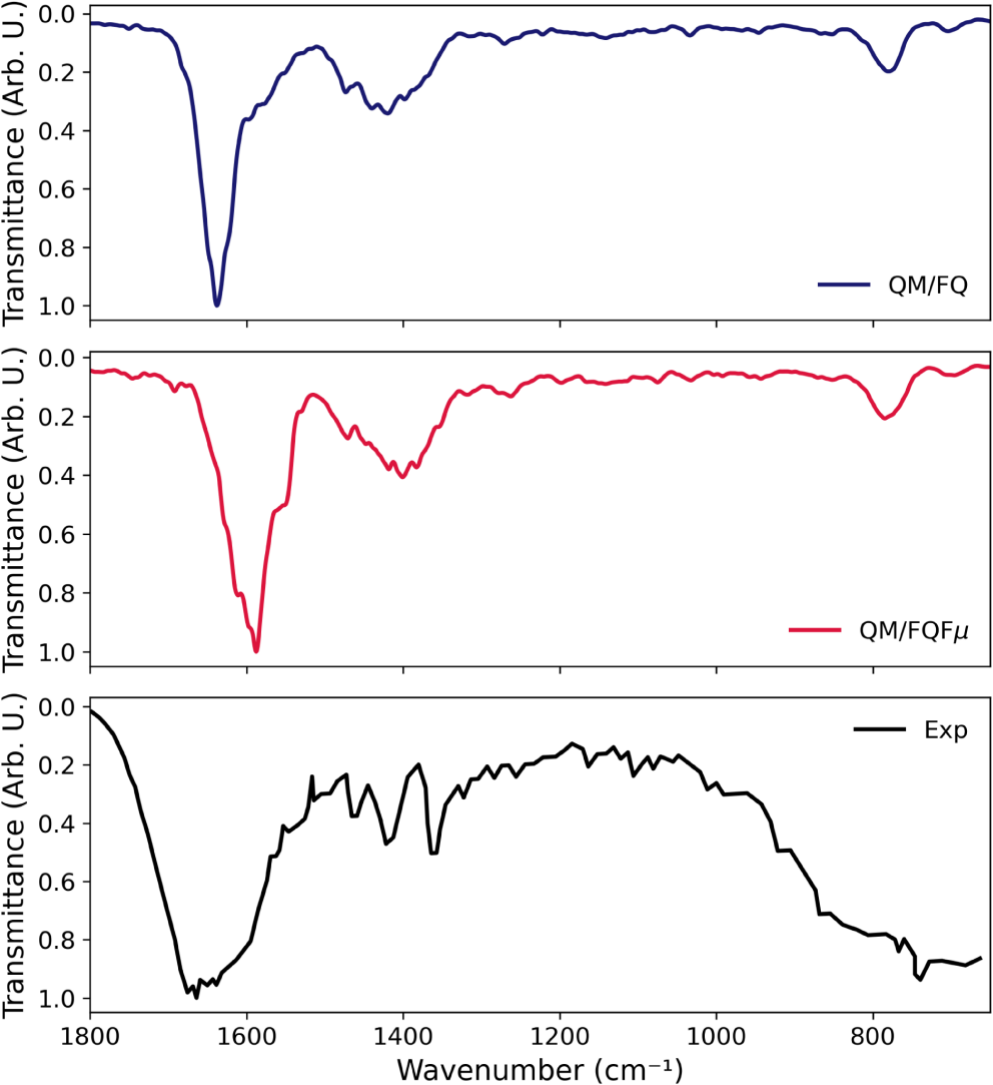
Computed IR spectra of l-tryptophan (TRP) in aqueous solution using QM/FQ and QM/FQFμ approaches at the B3LYP/TZ2P level of theory, averaged over 200 snapshots. The experimental IR spectrum^94^ is also shown for comparison.

In addition to these main bands, several weaker features appear in the 1300–900 cm^–1^ region, which arise from combinations of in-plane and out-of-plane bending modes of aromatic and aliphatic C–H groups, along with C–C and C–N stretching modes. The rocking of CH_2_ and ${\mathrm{NH}}_{3}^{+}$ groups give minor contributions to this region. As illustrated in Figure 9, IR spectra obtained with the QM/FQ and QM/FQFμ methods show minimal differences. The only noticeable deviation concerns the dominant band assigned to the asymmetric stretching of the carboxylate ion: it is slightly red-shifted in the QM/FQFμ spectrum and appears broader and better resolved than at the QM/FQ level. Apart from this minor difference, all remaining bands coincide in both positions and intensities, yielding two essentially identical spectra.

The computed Raman spectrum at 634 nm is reported in Figure 10. The dominant vibrational features substantially overlap with those observed in the IR spectrum. The band located at 1460 cm^–1^ primarily involves symmetric stretching of the carboxylate group, coupled with aliphatic C–C stretching and out-of-plane C–H bending. The most intense band, located at 1360 cm^–1^, is mainly attributed to aromatic C–C stretching, with additional contributions from in-plane C–H bending. Bands around 1200 cm^–1^ and 900 cm^–1^ are associated with mixed in-plane and out-of-plane bending, rocking, and stretching of aliphatic moieties. In particular, the intense band at approximately 1050 cm^–1^ is associated with C–C and C–N stretching in combination with in plane aromatic C–H bending modes.

**Figure 10:**
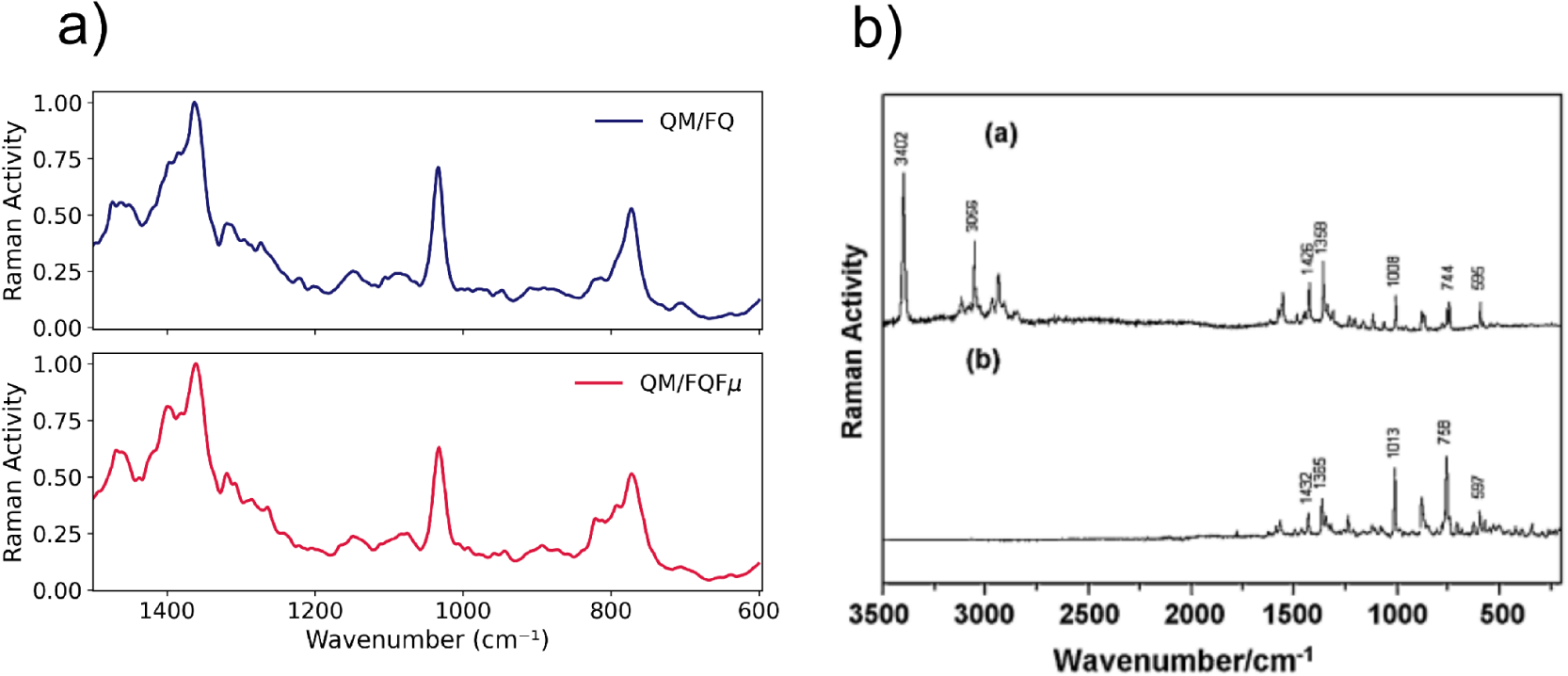
a) Computed Raman spectra of l-tryptophan (TRP) in aqueous solution using QM/FQ and QM/FQFμ approaches at the B3LYP/TZ2P level of theory, averaged over 200 snapshots. b) Experimental Raman spectra. Reproduced with permission from ref.^94^ Copyright Elsevier 2012. The top panel corresponds to the solid phase, while the bottom panel shows the experimental Raman spectrum of zwitterionic l-tryptophan in aqueous solution, used here for comparison with our results.

Finally, the 780 cm^–1^ band, which is also observed in the IR spectrum, includes contributions from CO_2_ deformation, aromatic out-of-plane bending, and rocking of CH_2_ and ${\mathrm{NH}}_{3}^{+}$ groups. Similarly to IR, the Raman spectra reported in Figure 10 reveal only subtle differences between the QM/FQ and QM/FQFμ approaches. The main vibrational bands are well-aligned in both positions and relative intensities, with the QM/FQFμ spectrum displaying slightly sharper features.

As expected, the ROA spectrum (Figure 11) exhibits a pronounced alternation of positive and negative signals, underscoring the intrinsic complexity of chiroptical responses. This spectral variability reflects both the conformational flexibility of the solute and the dynamic nature of solvent organization, as sampled by MD simulations. The major ROA bands correspond closely to those in the IR and Raman spectra, with the most intense signal assigned to the asymmetric stretching of the carboxylate group, consistently exhibiting a negative sign. Despite the intrinsic complexity of the ROA spectrum, the results reported in Figure 11 show a high level of consistency between the QM/FQ and QM/FQFμ models. Both approaches yield comparable band positions and reproduce the same sign pattern across the spectral range. In contrast to IR and Raman spectra—where the overall spectral shape remains essentially unchanged regardless of the number of snapshots, even though peak intensities and positions may vary—the ROA spectrum exhibits markedly slower convergence. As illustrated in Figures S15 and S18, using a limited number of snapshots can lead not only to intensity variations but also to incorrect sign predictions for key bands. This behavior further highlights the sensitivity of ROA signals to subtle conformational and environmental effects, reinforcing the notion that reliable chiroptical predictions require extensive configurational sampling. Similar predictive challenges have also been reported in previous studies. For instance, Blanch et al. demonstrated that the sign of the ROA band associated with the W3 vibration of the tryptophan indole ring (1550 cm^–1^) is highly sensitive to the local stereochemistry and conformation of the side chain, leading to sign inversions across different protein environments.^124^

**Figure 11:**
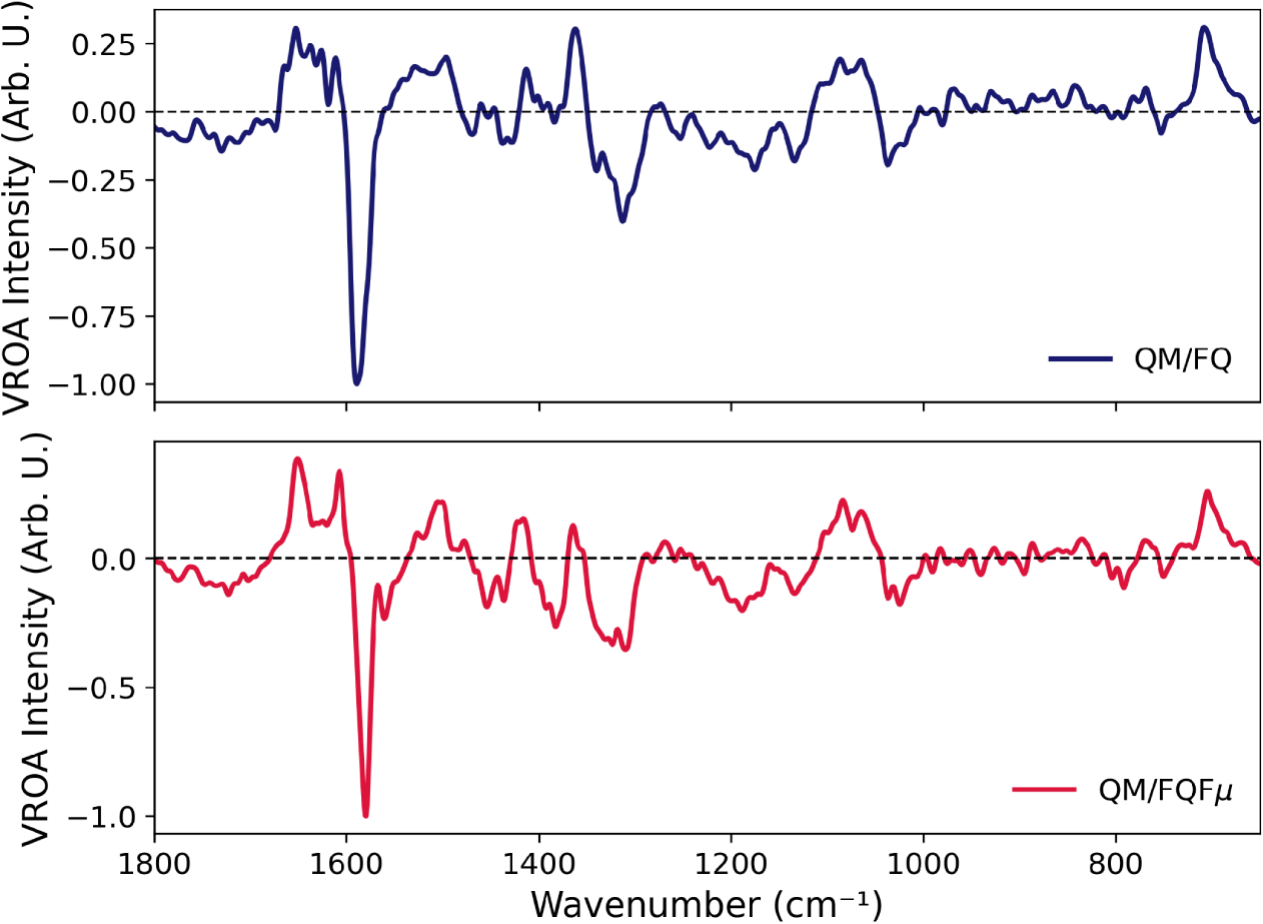
Computed ROA spectra of l-tryptophan (TRP) in aqueous solution using QM/FQ and QM/FQFμ approaches at the B3LYP/TZ2P level of theory, averaged over 200 snapshots.

We now move on to compare the computed results with the experimental data,^94^ which are available only for IR and Raman spectra. Computed IR spectra obtained with both QM/FQ and QM/FQFμ approaches are in good agreement with the experimental spectrum reported in the literature.^94^ The most intense band, corresponding to the asymmetric stretching of the carboxylate group, is centered around 1600 cm^–1^ in both theoretical and experimental spectra, confirming the reliability of the simulations in capturing the dominant vibrational features. However, while both models slightly underestimate the width of the experimental band, the QM/FQFμ spectrum shows a broader and smoother profile, which is more similar to the experimental shape.

The band centered near 1400 cm^–1^ in the simulated spectra also well reproduces the experimental profile. This region spans approximately from 1450 to 1350 cm^–1^ and includes multiple resolved components, while in the computed spectra it appears as a broad band.

The experimental spectral region between 1300 and 900 cm^–1^ is overall well reproduced by both computational approaches, with consistent band positions and relative intensities. The low-frequency region around 780 cm^–1^, which is associated with out-of-plane bending and rocking motions, is also present in the experimental spectrum, though shifted to lower wavenumbers (744 cm^–1^) and appearing with higher intensity. Despite the slight redshift, the computed band profile in this region is in good qualitative agreement with the experimental shape.

Simulated Raman spectra, both QM/FQ and QM/FQFμ, are in excellent agreement with experiments. The first band in the experimental spectrum, located at 1420 cm^–1^, aligns well with the simulated bands. However, a key difference lies in the intensity: the experimental band is weaker, whereas in the simulated spectrum, this band is the most intense.

Similarly, the band at 1360 cm^–1^ is present in both the experimental and computed spectra, with calculations accurately reproducing both the experimental band position and intensity.

Regarding the experimental bands at 1013 cm^–1^ and 758 cm^–1^, both are well reproduced in the computed spectra, both in terms of position and intensity. These bands are among the most intense in the experimental Raman spectrum, and the simulations accurately reflect these features.

## 4 Conclusions

In this work, we have applied multiscale modeling^12–15^ techniques to compute a broad range of spectroscopic properties—electronic, magnetic, and vibrational—of zwitterionic l-tryptophan in aqueous solution. The solute is treated at a high level of theory using density functional theory (DFT), while solute–solvent interactions, including hydrogen bonding, are captured through atomistic polarizable embedding models, QM/FQ and QM/FQFμ.^24,32,37,38^ To account for the inherently dynamic nature of both solute and solvent in solution, extensive sampling is performed via classical molecular dynamics simulations by using GROMACS package.^75^

We demonstrate that our computational protocol is not limited to a single spectroscopic observable but is broadly applicable across a variety of techniques. In particular, we have successfully simulated UV–vis and ECD spectra, OR values, NMR, IR, Raman, and ROA spectra. The UV spectroscopy results confirm that the bright S_0_ → S_1_ transition of tryptophan, which is observed experimentally^109^ at 278 nm, is accurately reproduced by both QM/FQ and QM/FQFμ approaches, with a deviation of approximately 14 nm. NBO analysis,^110,111^ performed on the representative QM/COSMO structure, confirms the π → π* character of the transition. The ECD spectrum is also successfully simulated, capturing the characteristic (−, +) sign pattern seen in the experimental data.^125^ As a chiroptical property, ECD is highly sensitive to the environment, and this is reflected in the more pronounced differences between the QM/FQ and QM/FQFμ models compared to UV spectra. In particular, the peak intensities in the ECD spectrum are better reproduced by the QM/FQFμ approach, underscoring the importance of an accurate treatment of the solvent in simulating such environment-dependent properties.

We then extend our investigation to another fundamental chiroptical observable: OR. As with ECD, OR is highly sensitive to both the molecular environment and conformational fluctuations. Remarkably, despite the large frame-by-frame variability observed throughout the molecular dynamics trajectory, both QM/FQ and QM/FQFμ models are able to correctly capture the experimental negative sign of the optical rotation at the sodium D-line. The computed values, −64 degree/(dm · g/cm^3^) for QM/FQ and −71 degree/(dm · g/cm^3^) for QM/FQFμ, show good agreement with the experimental measurement of −31.5° at 23°C.^116^

To further broaden the scope of the analysis, magnetic properties are also investigated through the simulation of NMR spectra using both QM/FQ and QM/FQFμ models. The chemical shifts of hydrogen and carbon nuclei are computed and compared with available experimental data^114,115^ while for nitrogen and oxygen atoms, only theoretical shielding values are obtained due to the lack of experimental references. For hydrogen nuclei, the coupled ^1^H NMR spectra are also simulated, including scalar spin–spin coupling constants. The resulting ^1^H and ^13^C spectra show good agreement with experiment, and only minor differences are observed between the two solvation models, indicating a relatively low sensitivity of these nuclei to the specific environment description. In contrast, more significant variations are found for the oxygen shielding constants, underscoring the importance of solvent treatment for accurately describing the magnetic responses of these nuclei.

Given the local nature of NMR observables and their strong dependence on the electronic density around each nucleus, a hybrid QM/FDE/FQFμ approach is also employed for this property.^35,37,38^ In this model, solvent molecules within a 3 Å radius of the solute are treated at the quantum level using a frozen density embedding (FDE) scheme, and freeze-and-thaw cycles are included to allow mutual polarization between the active and frozen regions. As expected, this refinement leads to a substantial improvement in the description of oxygen shielding, while only modest changes are observed for hydrogen and carbon, further confirming their lower sensitivity to the environment.

We also investigate vibrational properties, including IR, Raman, and ROA spectra, by applying QM/FQ and QM/FQFμ models, further confirming the general applicability of our protocol across a wide range of spectroscopic techniques. These spectroscopic techniques provide detailed insights into the vibrational structure and enable the characterization of both achiral and chiroptical responses. The analysis of the computed vibrational modes reveals that the main normal modes involved span a broad frequency range: at higher frequencies (1700–1300 cm^–1^), we observe the symmetric and asymmetric stretching of the carboxylate group, C=C stretching, ${\mathrm{NH}}_{3}^{+}$ bending, and in-plane C–H bending. At lower frequencies (1300–700 cm^–1^), the main contributions come from single bond stretching modes (C–C and C–N) and out-of-plane bending motions involving C–H and the carboxylate group. IR and Raman spectra are consistently reproduced by both QM/FQ and QM/FQFμ models, with only minor differences observed between them. For these two techniques, the computed results show good agreement with experimental data.^94^ ROA spectra likewise show good consistency between the two models, particularly in terms of band positions and sign patterns; however, due to the absence of experimental ROA data for zwitterionic tryptophan in aqueous solution, we cannot verify whether the predicted sign alternation is accurate.

## Supplementary Material



## Notes

TRP configurations in aqueous solution, TRP dihedral distribution functions, convergence plots of spectroscopic properties, NBO analysis, computed TRP NMR chemical shifts, shieldings, and coupling constants, absolute differences between chemical shifts and shieldings obtained with different solvent models, study of FDE parameters, experimental NMR H and C chemical shifts, IR, Raman, and ROA stick spectra, and TRP normal modes. (PDF)
