# Supplementary material for: Fully Atomistic Modeling in Computational Spectroscopy: Tryptophan in Aqueous Solution as a Test Case
Source: J Phys Chem A. 2025 Oct 30;129(45):10323–38. doi: 10.1021/acs.jpca.5c04511 (PMC12621259; doi:10.1021/acs.jpca.5c04511)
Supplement: Supplementary file 1 [file jp5c04511_si_001.pdf]

Supporting Information for: Fully Atomistic Modeling in Computational Spectroscopy: Tryptophan in Aqueous Solution as a Test Case

Marco Trinari,[†] Chiara Sepali,[†] Tommaso Giovannini,[‡] and Chiara Cappelli*[,†]

†*Scuola Normale Superiore, Piazza dei Cavalieri 7, I-56126 Pisa, Italy.*

‡*Department of Physics, University of Rome Tor Vergata, and INFN, Via della Ricerca Scientifica 1, I-00133, Rome, Italy*

E-mail: chiara.cappelli@sns.it

S1 L-tryptophan configurations in aqueous solution

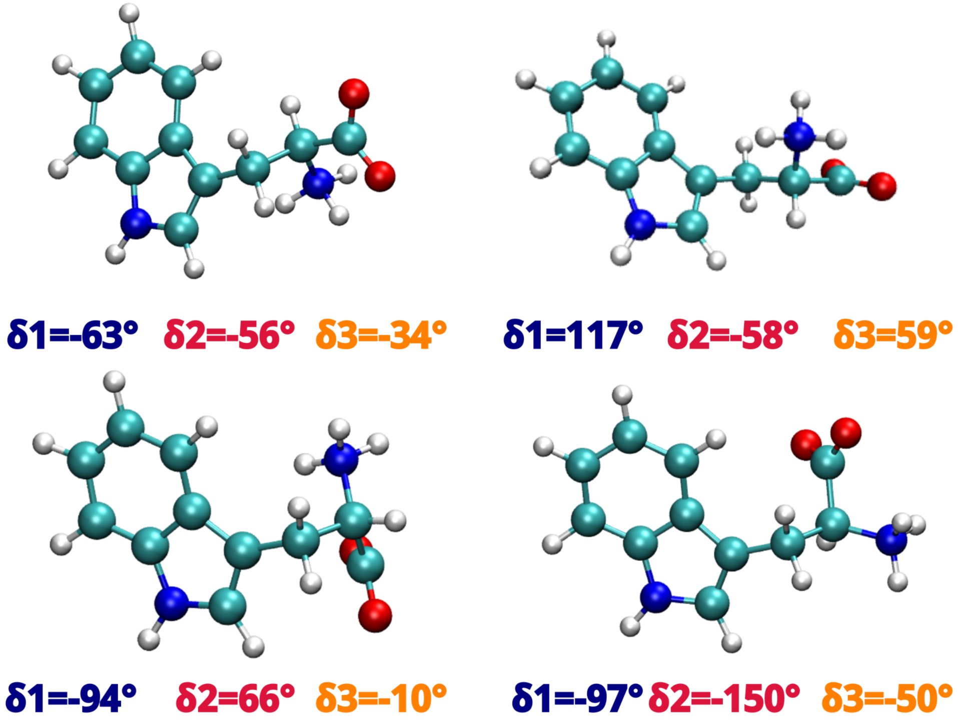

Figure S1: Representative structures of the four main conformers of L-tryptophan (TRP) in aqueous solution, along with the values of the three dihedral angles (δ_1, δ_2, and δ_3).

S2 Dihedral Distribution Functions

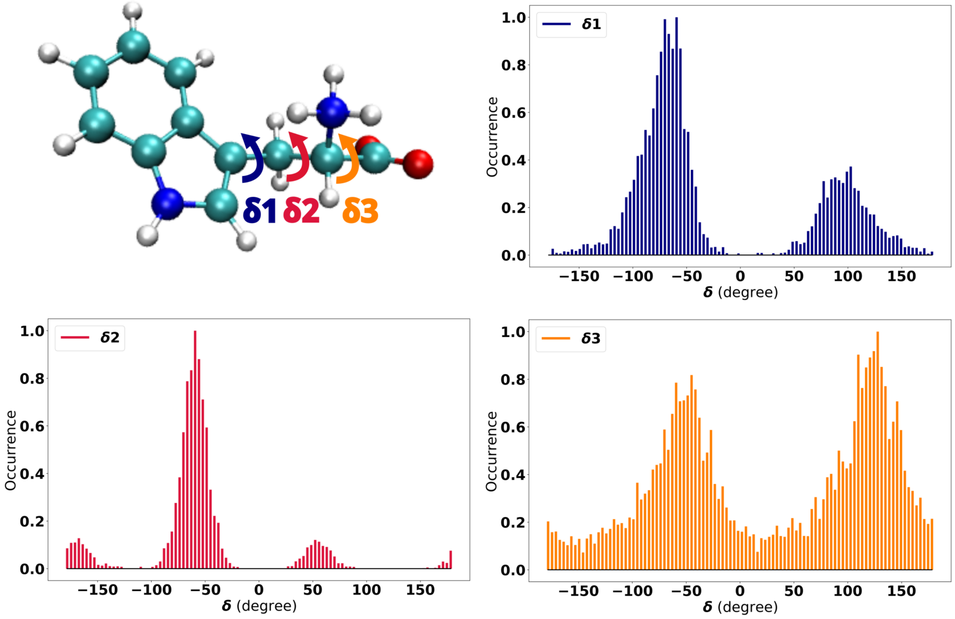

Figure S2: Definition of the L-Tryptophan dihedral angles δ_1, δ_2, and δ_3, and their corresponding dihedral distribution functions (DDFs).

S3 UV and ECD spectra

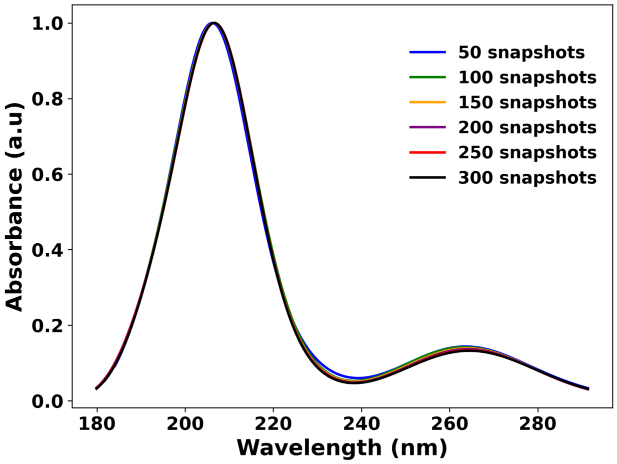

Figure S3: QM/FQ UV spectra of L-tryptophan in aqueous solution obtained by averaging a varying number of snaphosts (from 50 to 300) extracted from the MD trajectory. The spectrum stabilizes after 200 snapshots, with no significant changes observed beyond this point.

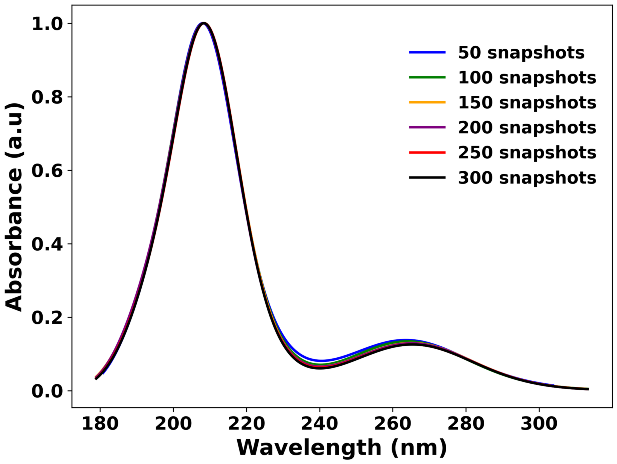

Figure S4: QM/FQFμ UV spectra of L-tryptophan in aqueous solution obtained by averaging a varying number of snaphosts (from 50 to 300) extracted from the MD trajectory. The spectrum stabilizes after 200 snapshots, with no significant changes observed beyond this point.

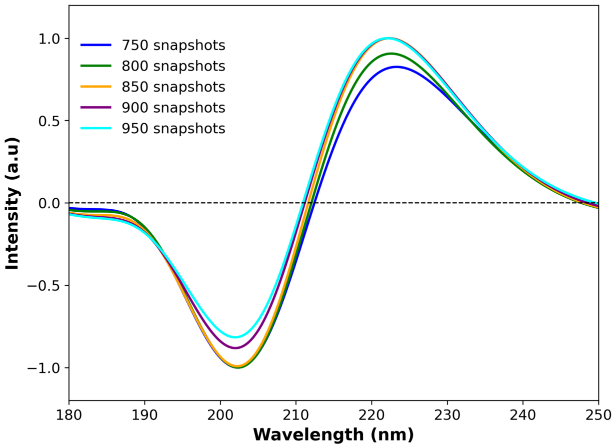

Figure S5: QM/FQ ECD spectra of L-tryptophan in aqueous solution obtained by averaging a varying number of snaphosts (from 750 to 950) extracted from the MD trajectory. The spectrum stabilizes after 900 snapshots, with no significant changes observed beyond this point.

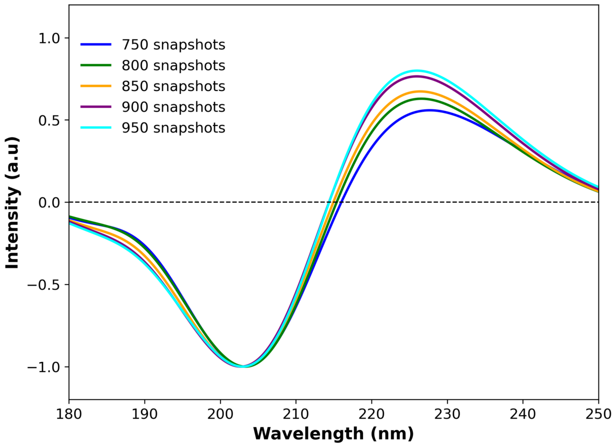

Figure S6: QM/FQFμ ECD spectra of L-tryptophan in aqueous solution obtained by averaging a varying number of snaphosts (from 750 to 950) extracted from the MD trajectory. The spectrum stabilizes after 900 snapshots, with no significant changes observed beyond this point.

S4 NBO analysis

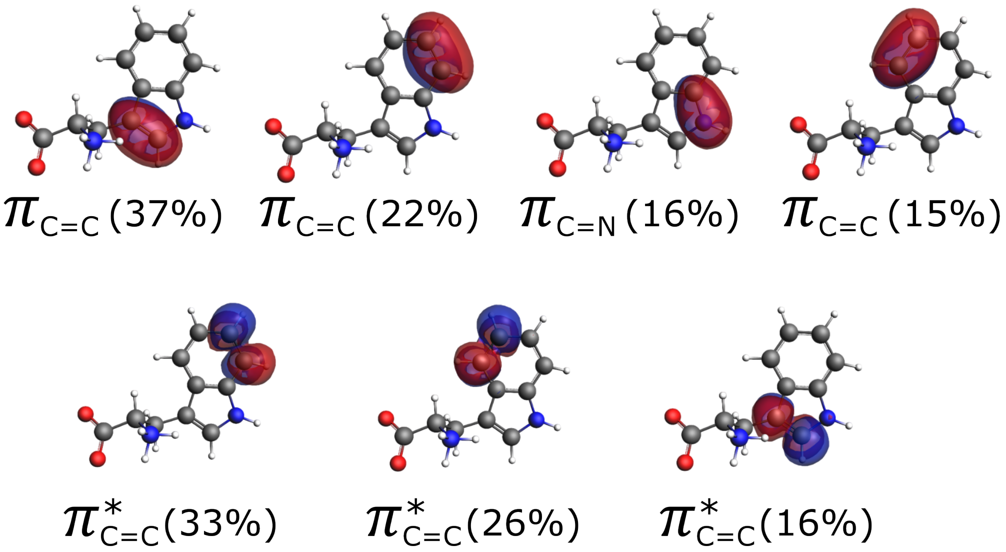

Figure S7: Orbitals involved in the main electronic transitions of solvated L-Tryptophan: the HOMO is a combination of the $\pi_{C=C}$ and $\pi_{C=N}$ orbitals whereas the LUMO is primarily composed of $\pi^*_{C=C}$ orbitals.

S5 NMR

Table S1: QM/FQ NMR computed chemical shifts (ppm) (for H and C nuclei) and shieldings (for O and N nuclei) of L-tryptophan in aqueous solution. Values are obtained by averaging a varying numbers of structures (from 150 to 200) extracted from the MD trajectory. Values stabilize after 150 snapshots, with no significant changes observed beyond this point.

**Atom**	**150**	**160**	**170**	**180**	**190**	**200**
H1	3.730	3.731	3.717	3.727	3.736	3.734
H2	3.446	3.443	3.440	3.439	3.445	3.438
H3	4.422	4.412	4.404	4.381	4.383	4.376
H4	9.994	9.984	9.981	9.983	10.005	10.006
H5	6.064	6.064	6.065	6.029	6.014	6.013
H6	5.981	5.971	5.961	5.932	5.930	5.928
H7	6.423	6.411	6.411	6.408	6.383	6.373
H8	8.211	8.205	8.203	8.200	8.201	8.204
H9	7.645	7.639	7.636	7.626	7.623	7.621
H10	7.785	7.793	7.792	7.784	7.782	7.778
H11	8.066	8.066	8.052	8.058	8.058	8.051
H12	7.957	7.955	7.948	7.953	7.962	7.972
C1	63.388	63.466	63.635	63.793	63.861	63.952
C2	30.670	30.655	30.642	30.580	30.576	30.437
C3	176.409	176.371	176.295	176.317	176.265	176.275
C4	117.642	117.652	117.793	117.816	117.827	117.763
C5	135.175	135.195	135.025	135.031	135.106	135.247
C6	143.749	143.806	143.933	143.962	143.931	143.871
C7	132.327	132.338	132.454	132.520	132.588	132.689
C8	124.408	124.385	124.429	124.438	124.420	124.387
C9	127.401	127.192	127.153	126.987	127.038	127.027
C10	129.827	129.835	129.653	129.649	129.598	129.451
C11	121.833	121.856	121.788	121.716	121.691	121.642
N1	210.360	210.459	210.455	210.395	210.429	210.498
N2	104.059	103.955	104.082	104.046	104.057	104.010
O1	34.471	34.977	34.551	35.017	35.489	35.161
O2	50.472	49.859	49.792	49.098	48.957	48.898

Table S2: QM/FQFμ NMR computed chemical shifts (ppm) (for H and C nuclei) and shieldings (ppm) (for O and N nuclei) of L-tryptophan in aqueous solution. Values are obtained by averaging a varying numbers of structures (from 150 to 200) extracted from the MD trajectory. Values stabilize after 150 snapshots, with no significant changes observed beyond this point.

**Atom**	**150**	**160**	**170**	**180**	**190**	**200**
H1	3.654	3.657	3.640	3.652	3.662	3.663
H2	3.496	3.490	3.487	3.481	3.485	3.474
H3	4.439	4.429	4.421	4.401	4.406	4.400
H4	10.397	10.384	10.383	10.379	10.400	10.402
H5	6.339	6.336	6.339	6.302	6.289	6.288
H6	6.251	6.240	6.224	6.195	6.191	6.188
H7	6.797	6.789	6.790	6.787	6.761	6.748
H8	8.250	8.244	8.244	8.242	8.242	8.245
H9	7.642	7.633	7.629	7.617	7.614	7.612
H10	7.802	7.808	7.806	7.795	7.791	7.786
H11	8.140	8.140	8.124	8.129	8.128	8.120
H12	8.052	8.050	8.042	8.045	8.055	8.064
C1	63.111	63.173	63.329	63.466	63.516	63.595
C2	30.692	30.679	30.673	30.621	30.607	30.464
C3	178.668	178.633	178.551	178.581	178.516	178.520
C4	117.181	117.160	117.291	117.314	117.321	117.252
C5	136.419	136.461	136.269	136.266	136.335	136.487
C6	143.667	143.725	143.864	143.900	143.864	143.812
C7	132.234	132.237	132.376	132.468	132.554	132.669
C8	124.538	124.535	124.617	124.647	124.624	124.588
C9	127.155	126.916	126.862	126.669	126.729	126.699
C10	129.650	129.657	129.453	129.430	129.358	129.179
C11	122.317	122.333	122.242	122.154	122.124	122.069
N1	211.602	211.718	211.734	211.668	211.719	211.768
N2	101.882	101.743	101.875	101.883	101.923	101.863
O1	41.320	41.855	41.481	42.068	42.575	42.176
O2	57.302	56.659	56.514	55.809	55.574	55.550

Table S3: Absolute differences (ppm) between chemical shifts (for H and C) and nuclear magnetic shieldings (ppm) (for N and O) computed using QM/FQ and QM/FQFμ, grouped by nucleus of L-tryptophan in aqueous solution.

**Atom**	**—QM/FQ - QM/FQFμ—**
**Hydrogen**	
H1	0.071
H2	0.036
H3	0.024
H4	0.396
H5	0.275
H6	0.261
H7	0.375
H8	0.041
H9	0.009
H10	0.008
H11	0.069
H12	0.093
**Average Δ (H)**	**0.138**
**Carbon**	
C1	0.356
C2	0.028
C3	2.245
C4	0.511
C5	1.241
C6	0.059
C7	0.020
C8	0.201
C9	0.328
C10	0.272
C11	0.427
**Average Δ (C)**	**0.517**
**Nitrogen**	
N1	1.270
N2	2.146
**Average Δ (N)**	**1.708**
**Oxygen**	
O1	7.016
O2	6.652
**Average Δ (O)**	**6.834**

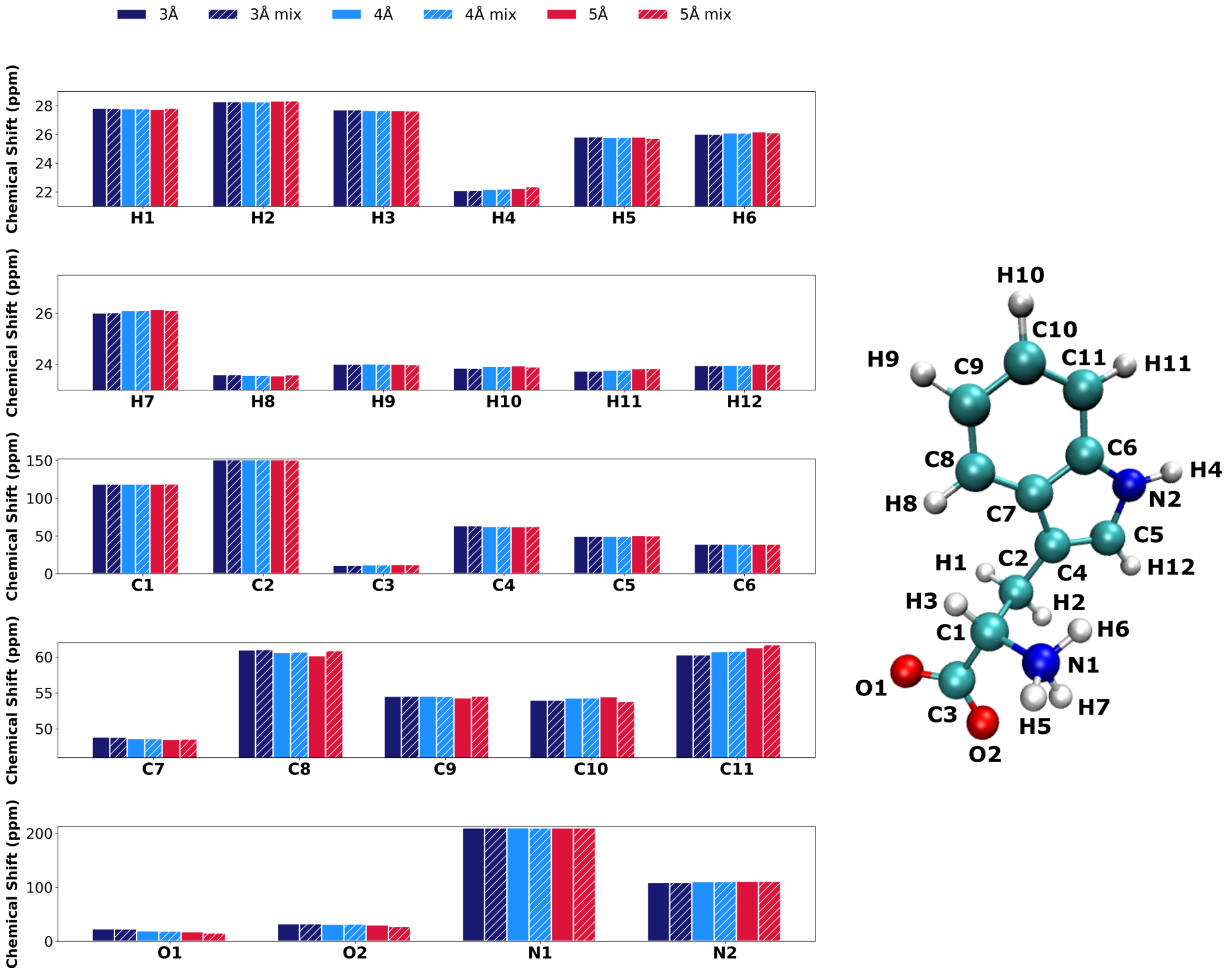

Figure S8: QM/FDE/FQFμ chemical shifts (ppm) (for H and C) and magnetic shieldings (ppm) (for O and N) computed for all atoms using different FDE spheres, with and without the use of a mixed basis set: TZ2P for the QM/FQFμ and QM/FDE/FQFμ steps, and DZP for the FDE/FQFμ step. The effect of increasing the FDE radius and changing the basis set is negligible for most atoms, with the exception of the hydrogen atoms bonded to nitrogen (H4, H5, H6, H7), the carboxylic carbon (C3) and the oxygen (O1,O2) and nitrogen (N1,N2) atoms.

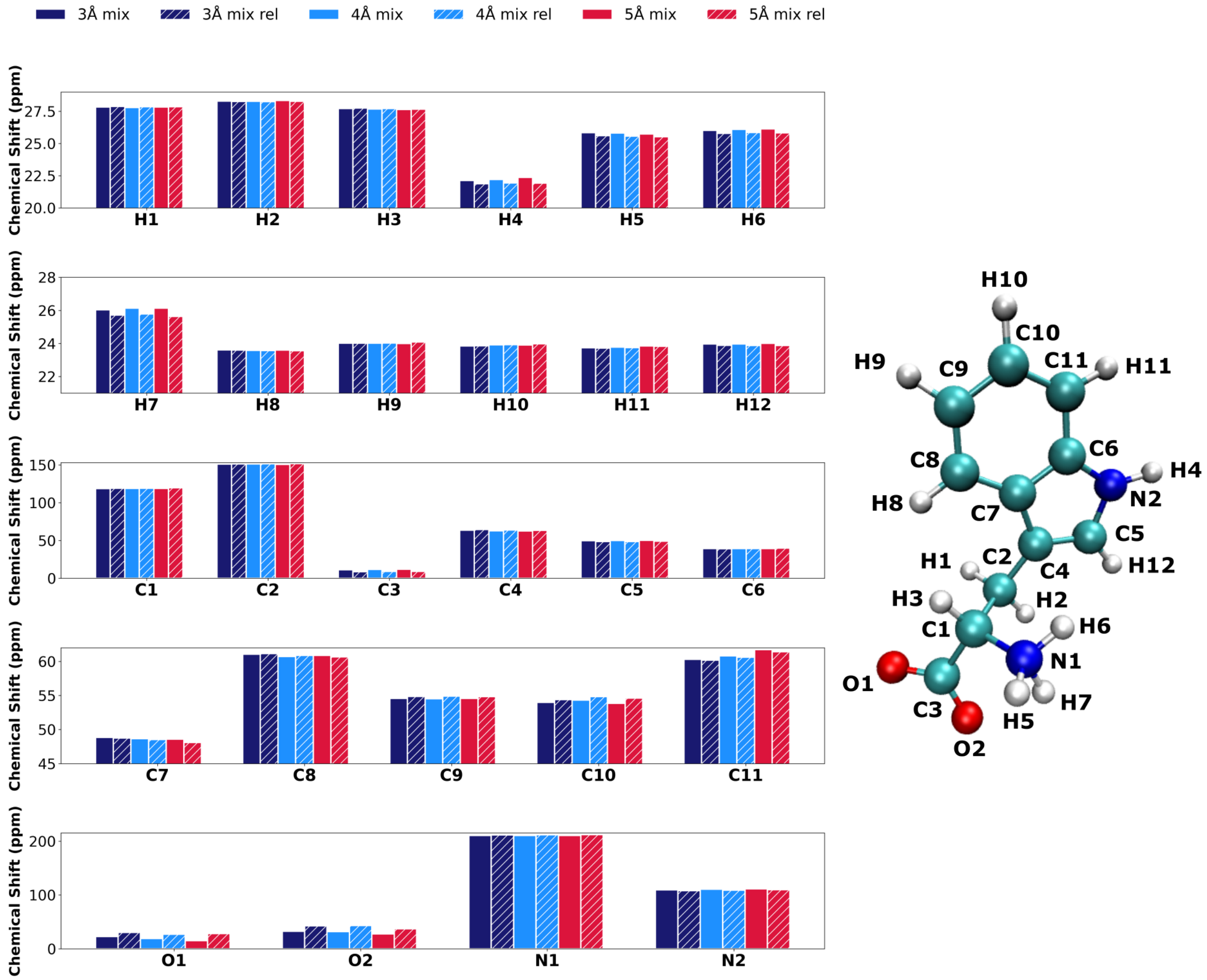

Figure S9: QM/FDE/FQFμ chemical shifts (ppm) (for H and C) and magnetic shieldings(ppm)(for O and N) computed for all atoms using different FDE radii, with and without the use of Freeze-and-thaw cycles. Including freeze-and-thaw cycles has little impact on most atoms, with notable exceptions observed for the hydrogen atoms bound to nitrogen (H4, H5, H6, H7), the carboxylic carbon (C3) and the oxygen (O1,O2) and nitrogen (N1,N2) atoms.

Table S4: QM/FDE/FQFμ NMR computed average chemical shifts (ppm) (for H and C nuclei) and shieldings (ppm) (for O and N nuclei) of L-tryptophan in aqueous solution, obtained with a varying number of structures (from 150 to 200) extracted from the MD trajectory. Values stabilize after 150 snapshots, with no significant changes observed beyond this point.

**Atom**	**150**	**160**	**170**	**180**	**190**	**200**
H1	3.713	3.719	3.706	3.712	3.719	3.719
H2	3.382	3.375	3.377	3.376	3.385	3.382
H3	4.344	4.341	4.334	4.314	4.318	4.310
H4	9.826	9.820	9.821	9.823	9.845	9.843
H5	5.920	5.914	5.908	5.881	5.866	5.864
H6	5.846	5.827	5.816	5.780	5.777	5.770
H7	6.234	6.231	6.240	6.234	6.214	6.203
H8	8.155	8.150	8.145	8.135	8.133	8.137
H9	7.646	7.636	7.633	7.624	7.624	7.625
H10	7.792	7.784	7.788	7.786	7.785	7.782
H11	8.063	8.057	8.047	8.056	8.060	8.057
H12	7.904	7.905	7.895	7.902	7.909	7.916
C1	63.567	63.650	63.852	64.016	64.090	64.206
C2	30.679	30.662	30.645	30.595	30.603	30.464
C3	174.315	174.269	174.200	174.225	174.203	174.205
C4	117.937	117.971	118.124	118.109	118.077	118.040
C5	134.388	134.422	134.237	134.264	134.336	134.415
C6	143.908	143.979	144.106	144.134	144.101	144.041
C7	132.451	132.522	132.603	132.646	132.684	132.776
C8	124.130	124.159	124.125	124.055	123.980	123.933
C9	127.666	127.419	127.377	127.233	127.336	127.364
C10	130.193	130.094	129.994	130.031	130.005	129.875
C11	121.964	121.929	121.911	121.879	121.904	121.903
N1	210.550	210.666	210.687	210.645	210.723	210.781
N2	105.025	104.878	104.984	104.960	104.924	104.914
O1	26.842	27.142	27.121	27.577	28.302	28.014
O2	40.988	40.531	40.083	39.552	39.330	39.385

Table S5: Computed average chemical shifts (ppm) (for H and C) and magnetic shielding constants (ppm) (for N and O) obtained using different computational models. All values are averaged over 200 snapshots and computed at the B3LYP/TZ2P level of theory. Three computational models are employed: QM/FQ, QM/FQFμ, and QM/FDE/FQFμ.

Atom	QM/FQ	QM/FQFμ	QM/FDE/FQFμ
**Hydrogen**			
H1	3.7336	3.6629	3.7194
H2	3.4384	3.4743	3.3824
H3	4.3763	4.3996	4.3101
H4	10.0063	10.4016	9.8430
H5	6.0134	6.2879	5.8644
H6	5.9277	6.1879	5.7700
H7	6.3728	6.7483	6.2028
H8	8.2038	8.2446	8.1367
H9	7.6214	7.6119	7.6250
H10	7.7782	7.7860	7.7816
H11	8.0512	8.1204	8.0570
H12	7.9716	8.0644	7.9159
**Carbon**			
C1	63.9518	63.5953	64.2063
C2	30.4370	30.4645	30.4642
C3	176.2754	178.5201	174.2049
C4	117.7629	117.2523	118.0402
C5	135.2466	136.4871	134.4151
C6	143.8707	143.8123	144.0412
C7	132.6885	132.6688	132.7757
C8	124.3865	124.5879	123.9328
C9	127.0266	126.6992	127.3639
C10	129.4505	129.1790	129.8752
C11	121.6423	122.0687	121.9026
**Nitrogen**			
N1	210.4982	211.7675	210.7810
N2	104.0095	101.8629	104.9142
**Oxygen**			
O1	35.1612	42.1765	28.0140
O2	48.8983	55.5504	39.3849

Table S6: Absolute differences between chemical shifts (ppm) (for H and C) and nuclear shieldings (ppm) (for N and O) of L-tryptophan in aqueous solution computed using QM/FQ, QM/FQFμ, and QM/FDE/FQFμ, grouped by nucleus.

Atom	QM/FQ - QM/FQFμ	QM/FQ - QM/FDE/FQFμ	QM/FQFμ - QM/FDE/FQFμ
**Hydrogen**			
H1	0.071	0.014	0.056
H2	0.036	0.056	0.092
H3	0.024	0.066	0.090
H4	0.396	0.163	0.558
H5	0.275	0.149	0.423
H6	0.261	0.158	0.418
H7	0.375	0.170	0.545
H8	0.041	0.067	0.109
H9	0.009	0.004	0.013
H10	0.008	0.004	0.004
H11	0.069	0.006	0.063
H12	0.093	0.056	0.148
**Mean Δ (H)**	**0.138**	**0.076**	**0.210**
**Carbon**			
C1	0.356	0.255	0.610
C2	0.028	0.027	0.001
C3	2.245	2.071	4.316
C4	0.511	0.277	0.788
C5	1.241	0.832	2.072
C6	0.059	0.171	0.229
C7	0.020	0.088	0.107
C8	0.201	0.454	0.655
C9	0.328	0.337	0.665
C10	0.272	0.424	0.696
C11	0.427	0.261	0.165
**Mean Δ (C)**	**0.517**	**0.472**	**0.937**
**Nitrogen**			
N1	1.270	0.283	0.987
N2	2.146	0.905	3.051
**Mean Δ (N)**	**1.708**	**0.594**	**2.019**
**Oxygen**			
O1	7.016	7.147	14.163
O2	6.652	9.513	16.165
**Mean Δ (O)**	**6.834**	**8.330**	**15.164**

Table S7: Computed coupling constants (Hz) between hydrogen atoms in L-tryptophan (TRP), obtained using the QM/FQ model at the hybrid B3LYP/TZ2P level of theory. Values are computed over 200 snapshots extracted from the MD trajectory.

Atom	H1	H2	H3	H4	H5	H6	H7	H8	H9	H10	H11	H12
H1	0.00	18.58	5.41	0.30	0.13	0.54	0.24	0.07	0.11	0.01	0.13	1.54
H2	18.58	0.00	11.47	0.07	0.44	0.32	0.35	0.09	0.04	0.03	0.10	1.05
H3	5.41	11.47	0.00	0.03	4.50	6.41	7.83	0.11	0.03	0.06	0.04	0.03
H4	0.30	0.07	0.03	0.00	0.06	0.06	0.06	0.46	0.05	0.06	0.09	1.58
H5	0.13	0.44	4.50	0.06	0.00	17.76	18.17	0.02	0.03	0.05	0.05	0.00
H6	0.54	0.32	6.41	0.06	17.76	0.00	17.67	0.02	0.02	0.05	0.05	0.01
H7	0.24	0.35	7.83	0.06	18.17	17.67	0.00	0.02	0.03	0.05	0.05	0.02
H8	0.07	0.09	0.11	0.46	0.02	0.02	0.02	0.00	7.55	0.13	1.36	0.26
H9	0.11	0.04	0.03	0.05	0.03	0.02	0.03	7.55	0.00	7.63	0.02	0.10
H10	0.01	0.03	0.06	0.06	0.05	0.05	0.05	0.13	7.63	0.00	8.09	0.43
H11	0.13	0.10	0.04	0.09	0.05	0.05	0.05	1.36	0.02	8.09	0.00	0.21
H12	1.54	1.05	0.03	1.58	0.00	0.01	0.02	0.26	0.10	0.43	0.21	0.00

Table S8: Computed coupling constants (Hz) between hydrogen atoms in L-tryptophan (TRP), obtained using the QM/FQFμ model at the hybrid B3LYP/TZ2P level of theory. Values are computed over 200 snapshots extracted from the MD trajectory.

Atom	H1	H2	H3	H4	H5	H6	H7	H8	H9	H10	H11	H12
H1	0.00	18.42	5.47	0.29	0.12	0.53	0.24	0.06	0.11	0.00	0.13	1.51
H2	18.42	0.00	11.57	0.07	0.44	0.32	0.35	0.09	0.04	0.03	0.10	1.04
H3	5.47	11.57	0.00	0.03	4.41	6.26	7.58	0.11	0.03	0.06	0.04	0.03
H4	0.29	0.07	0.03	0.00	0.06	0.06	0.06	0.45	0.05	0.06	0.09	1.59
H5	0.12	0.44	4.41	0.06	0.00	17.68	18.04	0.02	0.03	0.05	0.05	0.00
H6	0.53	0.32	6.26	0.06	17.68	0.00	17.59	0.03	0.02	0.05	0.05	0.01
H7	0.24	0.35	7.58	0.06	18.04	17.59	0.00	0.02	0.03	0.05	0.05	0.02
H8	0.06	0.09	0.11	0.45	0.02	0.03	0.02	0.00	7.55	0.14	1.35	0.26
H9	0.11	0.04	0.03	0.05	0.03	0.02	0.03	7.55	0.00	7.61	0.00	0.10
H10	0.00	0.03	0.06	0.06	0.05	0.05	0.05	0.14	7.61	0.00	8.10	0.42
H11	0.13	0.10	0.04	0.09	0.05	0.05	0.05	1.35	0.00	8.10	0.00	0.20
H12	1.51	1.04	0.03	1.59	0.00	0.01	0.02	0.26	0.10	0.42	0.20	0.00

Table S9: Computed coupling constants (Hz) between hydrogen atoms in L-tryptophan (TRP), obtained using the QM/FDE/FQFμ model at the hybrid B3LYP/TZ2P level of theory. Values are computed over 200 snapshots extracted from the MD trajectory.

Atom	H1	H2	H3	H4	H5	H6	H7	H8	H9	H10	H11	H12
H1	0.00	18.48	5.32	0.31	0.14	0.53	0.24	0.07	0.11	0.02	0.14	1.53
H2	18.48	0.00	11.37	0.07	0.44	0.31	0.34	0.10	0.04	0.02	0.11	1.06
H3	5.32	11.37	0.00	0.03	4.59	6.52	7.98	0.10	0.03	0.06	0.04	0.03
H4	0.31	0.07	0.03	0.00	0.06	0.06	0.06	0.47	0.06	0.06	0.10	1.57
H5	0.14	0.44	4.59	0.06	0.00	17.77	18.26	0.02	0.03	0.05	0.05	0.00
H6	0.53	0.31	6.52	0.06	17.77	0.00	17.65	0.02	0.02	0.05	0.05	0.01
H7	0.24	0.34	7.98	0.06	18.26	17.65	0.00	0.02	0.03	0.05	0.05	0.02
H8	0.07	0.10	0.10	0.47	0.02	0.02	0.02	0.00	7.55	0.12	1.37	0.26
H9	0.11	0.04	0.03	0.06	0.03	0.02	0.03	7.55	0.00	7.65	0.04	0.10
H10	0.02	0.02	0.06	0.06	0.05	0.05	0.05	0.12	7.65	0.00	8.10	0.43
H11	0.14	0.11	0.04	0.10	0.05	0.05	0.05	1.37	0.04	8.10	0.00	0.22
H12	1.53	1.06	0.03	1.57	0.00	0.01	0.02	0.26	0.10	0.43	0.22	0.00

Table S10: Absolute differences (Hz) between J-coupling constants computed using QM/FQ and QM/FQFμ models.

Atom	H1	H2	H3	H4	H5	H6	H7	H8	H9	H10	H11	H12
H1	0.000	0.160	0.060	0.010	0.010	0.010	0.000	0.010	0.000	0.010	0.000	0.030
H2	0.160	0.000	0.100	0.000	0.000	0.000	0.000	0.000	0.000	0.000	0.000	0.010
H3	0.060	0.100	0.000	0.000	0.090	0.150	0.250	0.000	0.000	0.000	0.000	0.000
H4	0.010	0.000	0.000	0.000	0.000	0.000	0.000	0.010	0.000	0.000	0.000	0.010
H5	0.010	0.000	0.090	0.000	0.000	0.080	0.130	0.000	0.000	0.000	0.000	0.000
H6	0.010	0.000	0.150	0.000	0.080	0.000	0.080	0.010	0.000	0.000	0.000	0.000
H7	0.000	0.000	0.250	0.000	0.130	0.080	0.000	0.000	0.000	0.000	0.000	0.000
H8	0.010	0.000	0.000	0.010	0.000	0.010	0.000	0.000	0.000	0.010	0.010	0.000
H9	0.000	0.000	0.000	0.000	0.000	0.000	0.000	0.000	0.000	0.020	0.020	0.000
H10	0.010	0.000	0.000	0.000	0.000	0.000	0.000	0.010	0.020	0.000	0.010	0.010
H11	0.000	0.000	0.000	0.000	0.000	0.000	0.000	0.010	0.020	0.010	0.000	0.010
H12	0.030	0.010	0.000	0.010	0.000	0.000	0.000	0.000	0.000	0.010	0.010	0.000

Table S11: Absolute differences (Hz) between J-coupling constants computed using QM/FQ and QM/FDE/FQ models.

**Atom**	**H1**	**H2**	**H3**	**H4**	**H5**	**H6**	**H7**	**H8**	**H9**	**H10**	**H11**	**H12**
H1	0.000	0.100	0.090	0.010	0.010	0.010	0.000	0.000	0.000	0.010	0.010	0.010
H2	0.100	0.000	0.100	0.000	0.000	0.010	0.010	0.010	0.000	0.010	0.010	0.010
H3	0.090	0.100	0.000	0.000	0.090	0.110	0.150	0.010	0.000	0.000	0.000	0.000
H4	0.010	0.000	0.000	0.000	0.000	0.000	0.000	0.010	0.010	0.000	0.010	0.010
H5	0.010	0.000	0.090	0.000	0.000	0.010	0.090	0.000	0.000	0.000	0.000	0.000
H6	0.010	0.010	0.110	0.000	0.010	0.000	0.020	0.000	0.000	0.000	0.000	0.000
H7	0.000	0.010	0.150	0.000	0.090	0.020	0.000	0.000	0.000	0.000	0.000	0.000
H8	0.000	0.010	0.010	0.010	0.000	0.000	0.000	0.000	0.000	0.010	0.010	0.000
H9	0.000	0.000	0.000	0.010	0.000	0.000	0.000	0.000	0.000	0.020	0.020	0.000
H10	0.010	0.010	0.000	0.000	0.000	0.000	0.000	0.010	0.020	0.000	0.010	0.000
H11	0.010	0.010	0.000	0.010	0.000	0.000	0.000	0.010	0.020	0.010	0.000	0.010
H12	0.010	0.010	0.000	0.010	0.000	0.000	0.000	0.000	0.000	0.000	0.010	0.000

Table S12: Absolute differences (Hz) between J-coupling constants computed using QM/FQFμ and QM/FDE/FQFμ models.

**Atom**	**H1**	**H2**	**H3**	**H4**	**H5**	**H6**	**H7**	**H8**	**H9**	**H10**	**H11**	**H12**
H1	0.000	0.060	0.150	0.020	0.020	0.000	0.000	0.010	0.000	0.020	0.010	0.020
H2	0.060	0.000	0.200	0.000	0.000	0.010	0.010	0.010	0.000	0.010	0.010	0.020
H3	0.150	0.200	0.000	0.000	0.180	0.260	0.400	0.010	0.000	0.000	0.000	0.000
H4	0.020	0.000	0.000	0.000	0.000	0.000	0.000	0.020	0.010	0.000	0.010	0.020
H5	0.020	0.000	0.180	0.000	0.000	0.090	0.220	0.000	0.000	0.000	0.000	0.000
H6	0.000	0.010	0.260	0.000	0.090	0.000	0.060	0.010	0.000	0.000	0.000	0.000
H7	0.000	0.010	0.400	0.000	0.220	0.060	0.000	0.000	0.000	0.000	0.000	0.000
H8	0.010	0.010	0.010	0.020	0.000	0.010	0.000	0.000	0.000	0.020	0.020	0.000
H9	0.000	0.000	0.000	0.010	0.000	0.000	0.000	0.000	0.000	0.040	0.040	0.000
H10	0.020	0.010	0.000	0.000	0.000	0.000	0.000	0.020	0.040	0.000	0.000	0.010
H11	0.010	0.010	0.000	0.010	0.000	0.000	0.000	0.020	0.040	0.000	0.000	0.020
H12	0.020	0.020	0.000	0.020	0.000	0.000	0.000	0.000	0.000	0.010	0.020	0.000

Table S13: Experimental chemical shifts (ppm) for hydrogen and carbon nuclei[1,2] of L-tryptophan in aqueous solution.

Nucleus	Experimental shift (ppm)
H1	3.292
H2	3.472
H3	4.046
H(aro)	7.194
H(aro)	7.274
H(aro)	7.310
H(aro)	7.531
H(aro)	7.723
C2	27.21
C1	56.10
C(aro)	108.74
C(aro)	112.74
C(aro)	119.27
C(aro)	120.27
C(aro)	122.93
C(aro)	125.76
C(aro)	127.69
C(aro)	137.28
C3	174.75

S6 OR

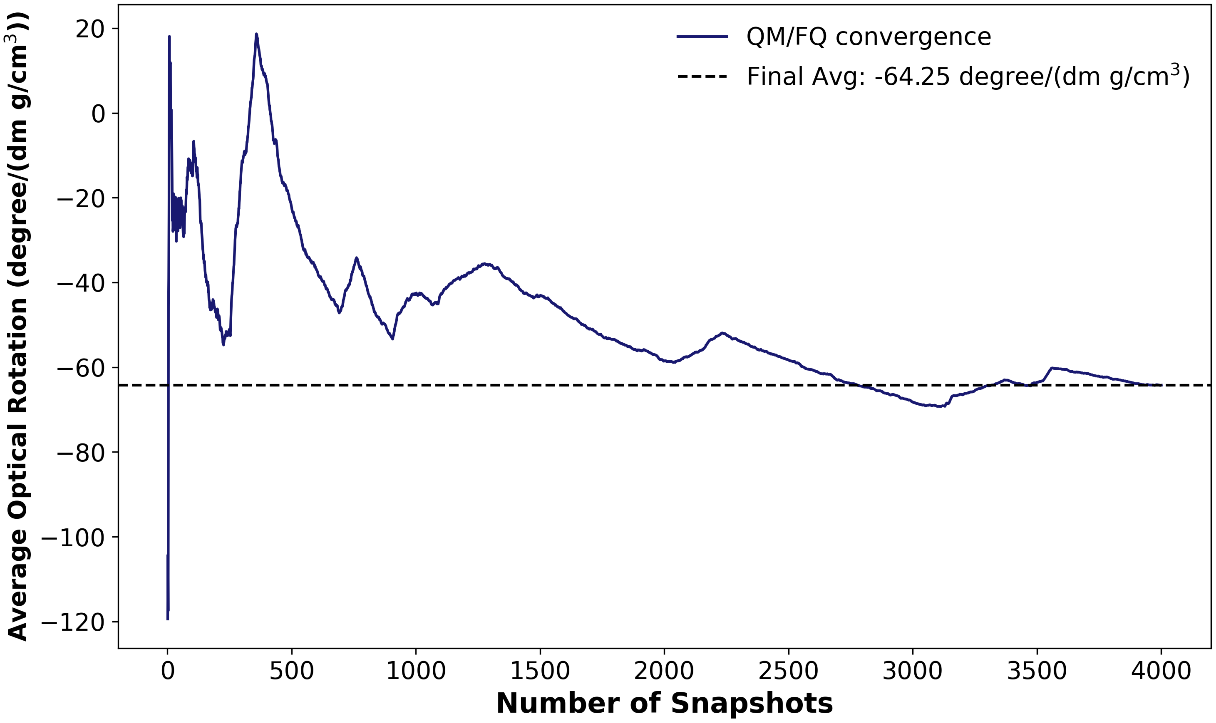

Figure S10: Convergence of the optical rotation of L-tryptophan in aqueous solution simulated with the QM/FQ model at the B3LYP/TZ2P level of theory. The average optical rotation at the sodium D-line (589.3 nm) is reported as a function of the number of MD frames included in the calculation. The dashed line indicates the final value obtained with 4000 frames. The plot highlights a progressive stabilization of the signal, with convergence reached after approximately 3300 frames, beyond which the values oscillate between -60 and -64 degree/(dm $\cdot$ g/cm^3).

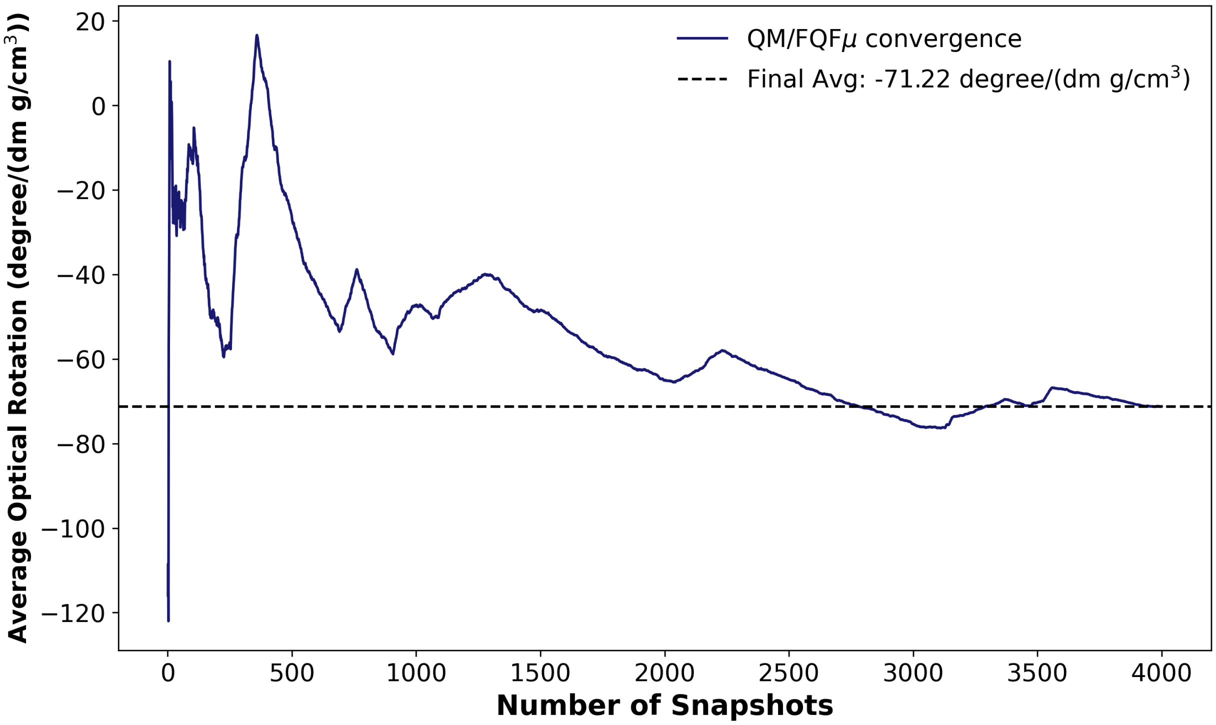

Figure S11: Convergence of the optical rotation of L-tryptophan in aqueous solution simulated with the QM/FQFμ model at the B3LYP/TZ2P level of theory. The average optical rotation at the sodium D-line (589.3 nm) is reported as a function of the number of MD frames included in the calculation. The dashed line indicates the final value obtained with 4000 frames. The plot highlights a progressive stabilization of the signal, with convergence reached after approximately 3300 frames, beyond which the values oscillate between -67 and -71 degree/(dm $\cdot$ g/cm^3).

S7 IR, Raman and ROA spectra

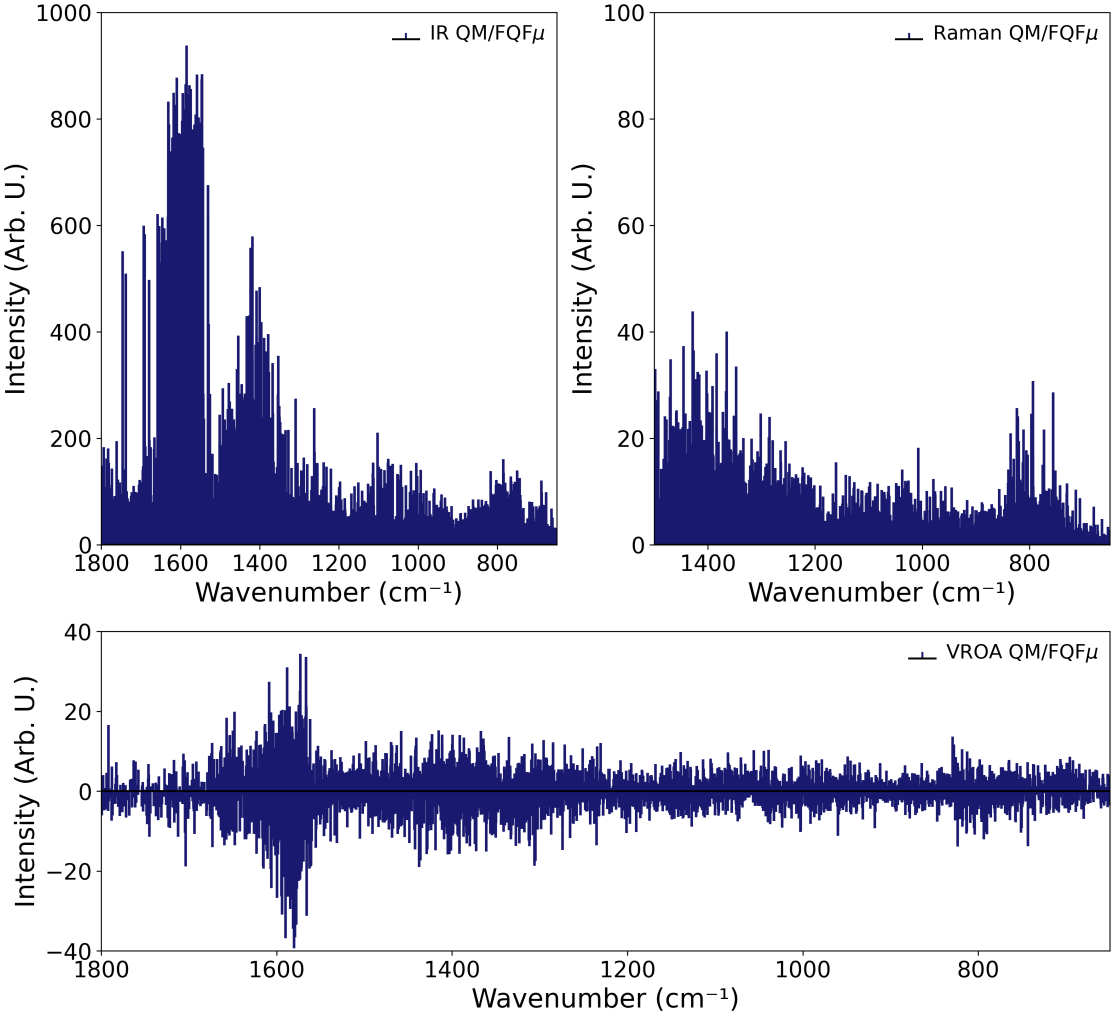

Figure S12: IR, Raman, and ROA stick spectra of L-tryptophan (TRP) in aqueous solution computed using the QM/FQFμ approach at the B3LYP/TZ2P level of theory. Spectra correspond to normal mode analyses performed on 230 individual snapshots extracted from the molecular dynamics simulation. IR and Raman spectra are shown in the top panel, and the ROA spectrum in the bottom panel.

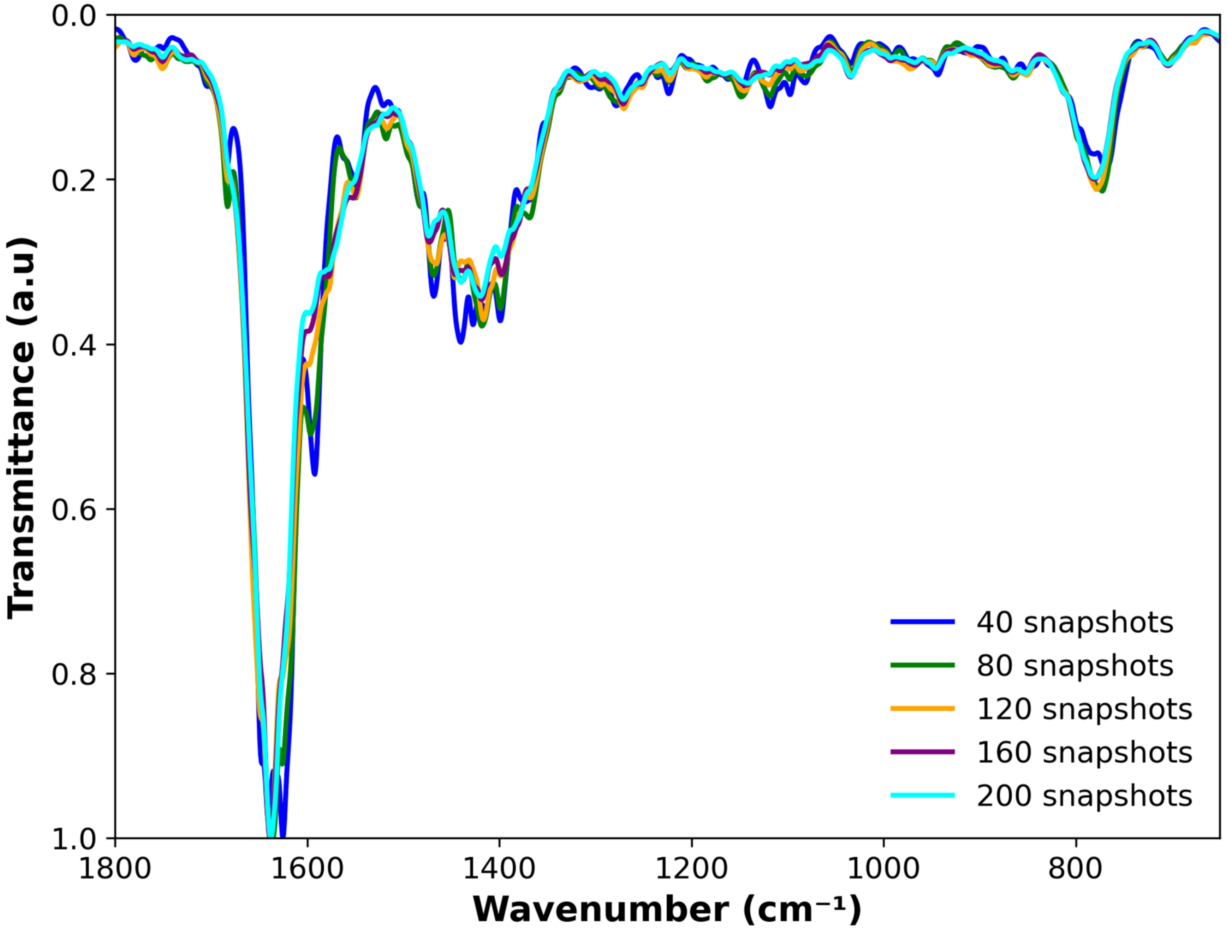

Figure S13: QM/FQ IR spectra of L-tryptophan in aqueous solution obtained with a varying numbers of structures (from 40 to 200) extracted from the MD trajectory. The spectrum stabilizes after 160 snapshots, with no significant changes observed beyond this point.

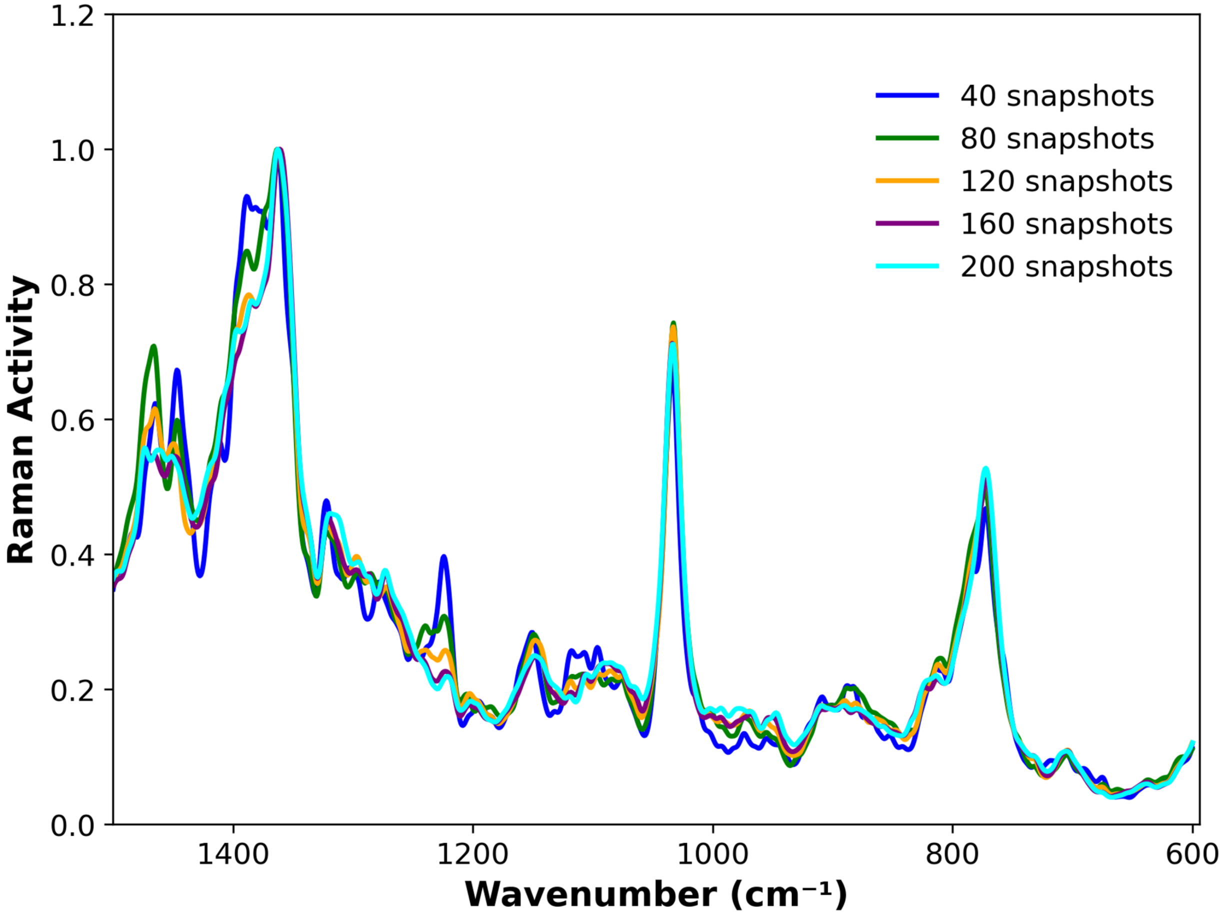

Figure S14: QM/FQ Raman spectra of L-tryptophan in aqueous solution obtained with a varying numbers of structures (from 40 to 200) extracted from the MD trajectory. The spectrum stabilizes after 160 snapshots, with no significant changes observed beyond this point.

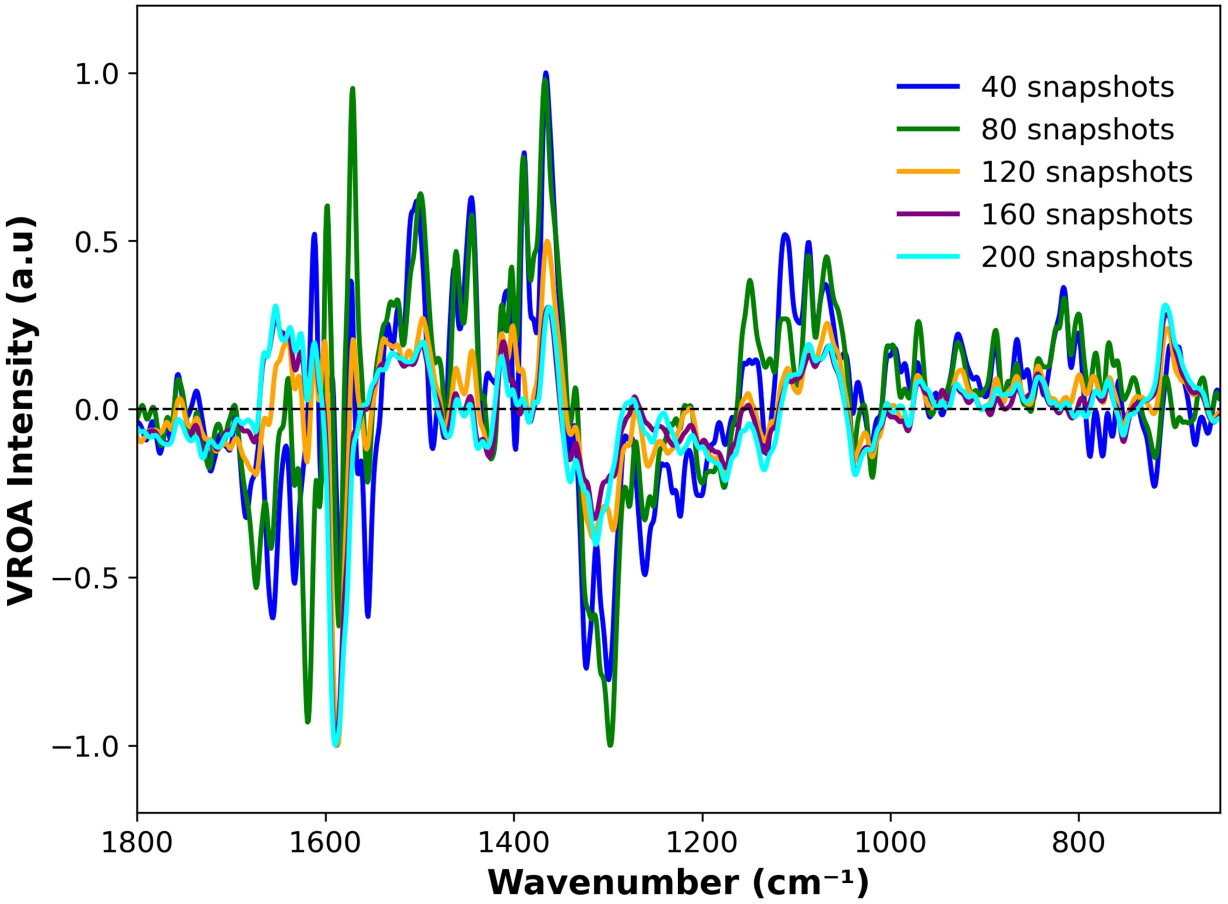

Figure S15: QM/FQ ROA spectra of L-tryptophan in aqueous solution obtained with a varying numbers of structures (from 40 to 200) extracted from the MD trajectory. The spectrum stabilizes after 160 snapshots, with no significant changes observed beyond this point.

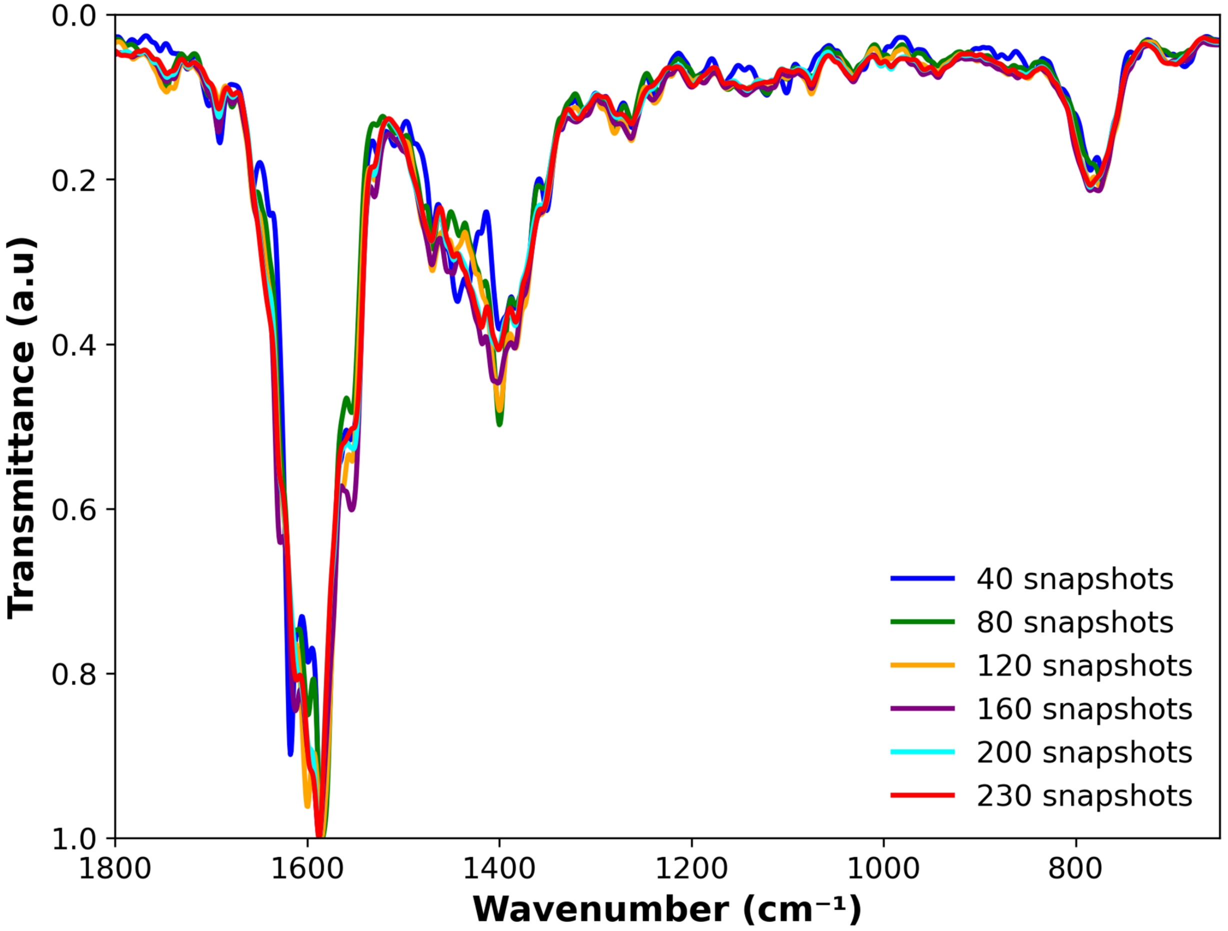

Figure S16: QM/FQFμ IR spectra of L-tryptophan in aqueous solution obtained with a varying numbers of structures (from 40 to 230) extracted from the MD trajectory. The spectrum stabilizes after 200 snapshots, with no significant changes observed beyond this point.

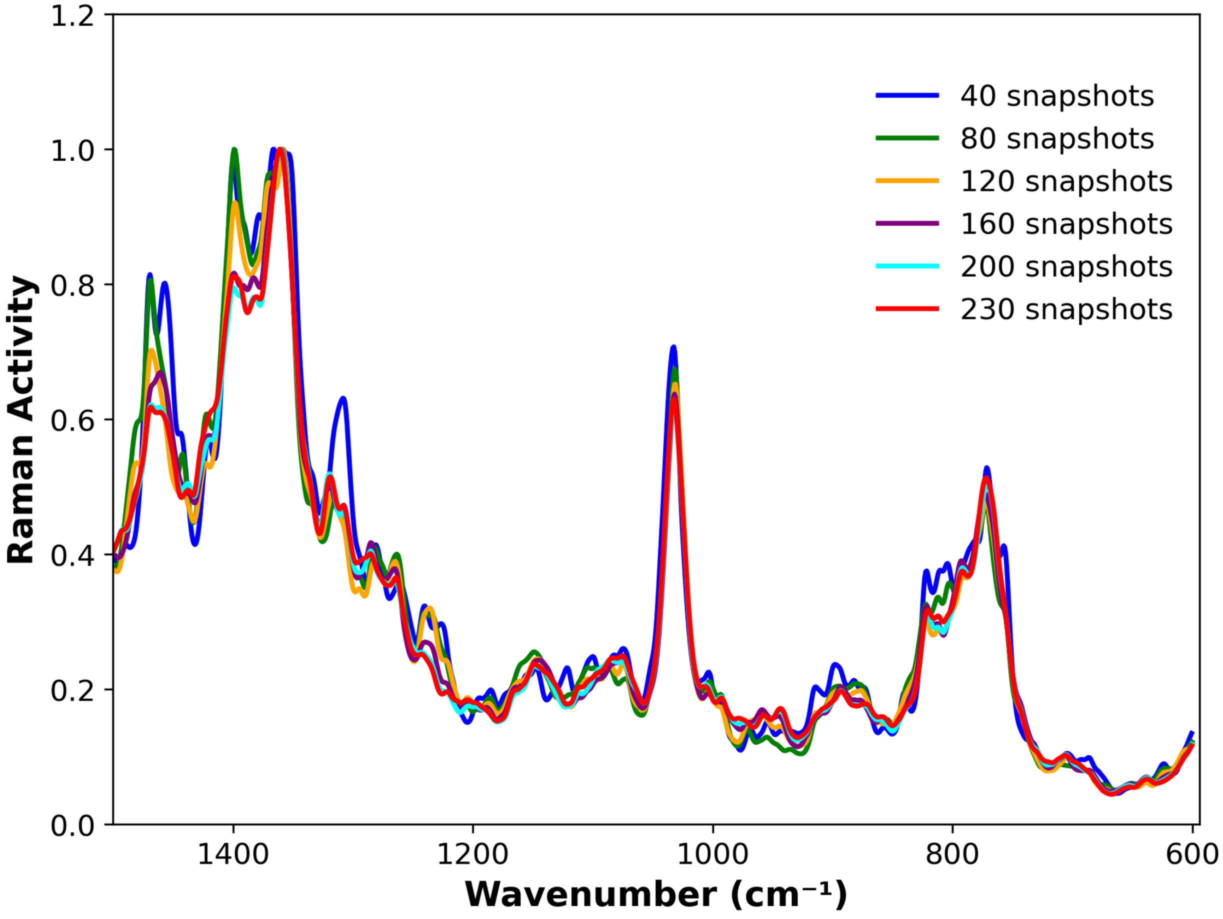

Figure S17: QM/FQFμ Raman spectra of L-tryptophan in aqueous solution obtained with a varying numbers of structures (from 40 to 230) extracted from the MD trajectory. The spectrum stabilizes after 200 snapshots, with no significant changes observed beyond this point.

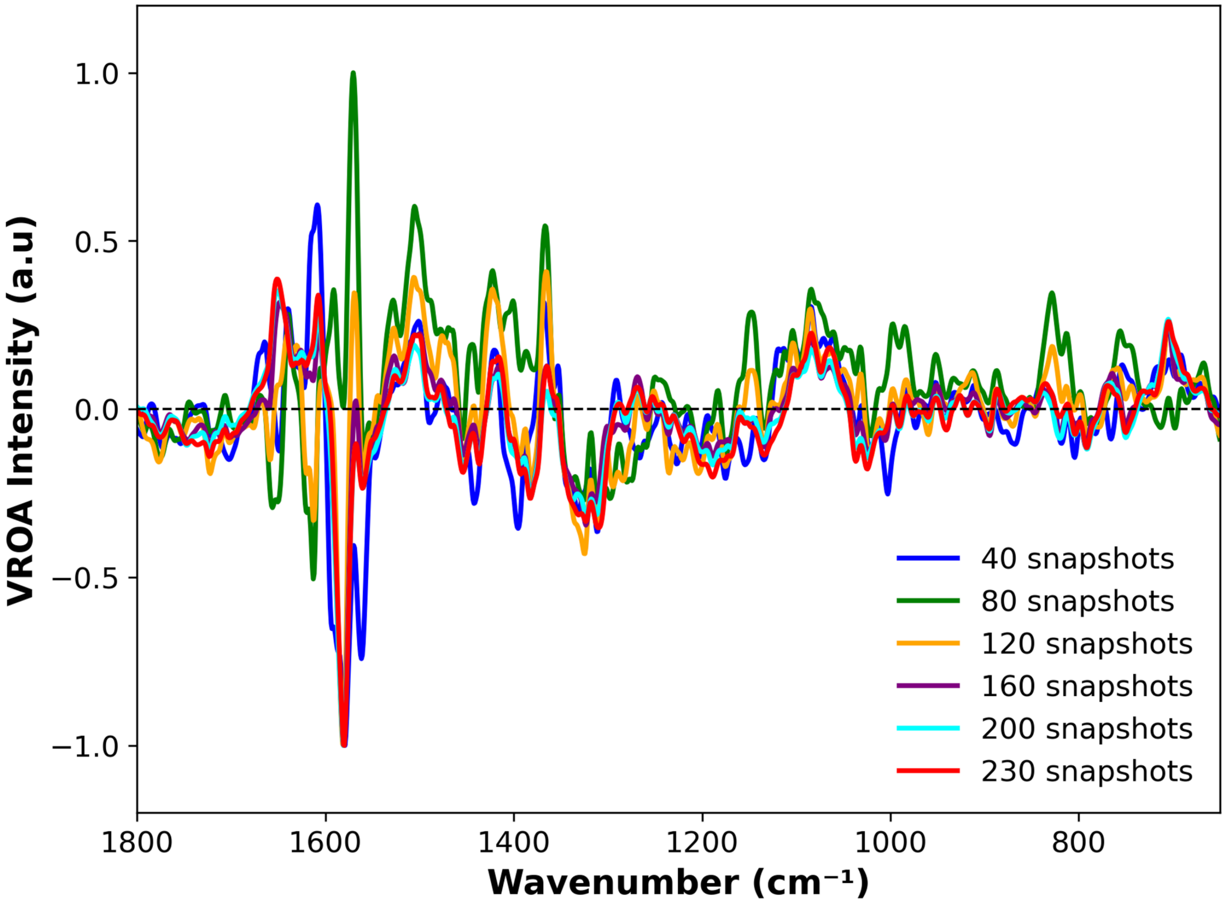

Figure S18: QM/FQFμ ROA spectra of L-tryptophan in aqueous solution obtained with a varying numbers of structures (from 40 to 230) extracted from the MD trajectory. The spectrum stabilizes after 200 snapshots, with no significant changes observed beyond this point.

S8 Normal Modes of L-tryptophan in aqueous solution

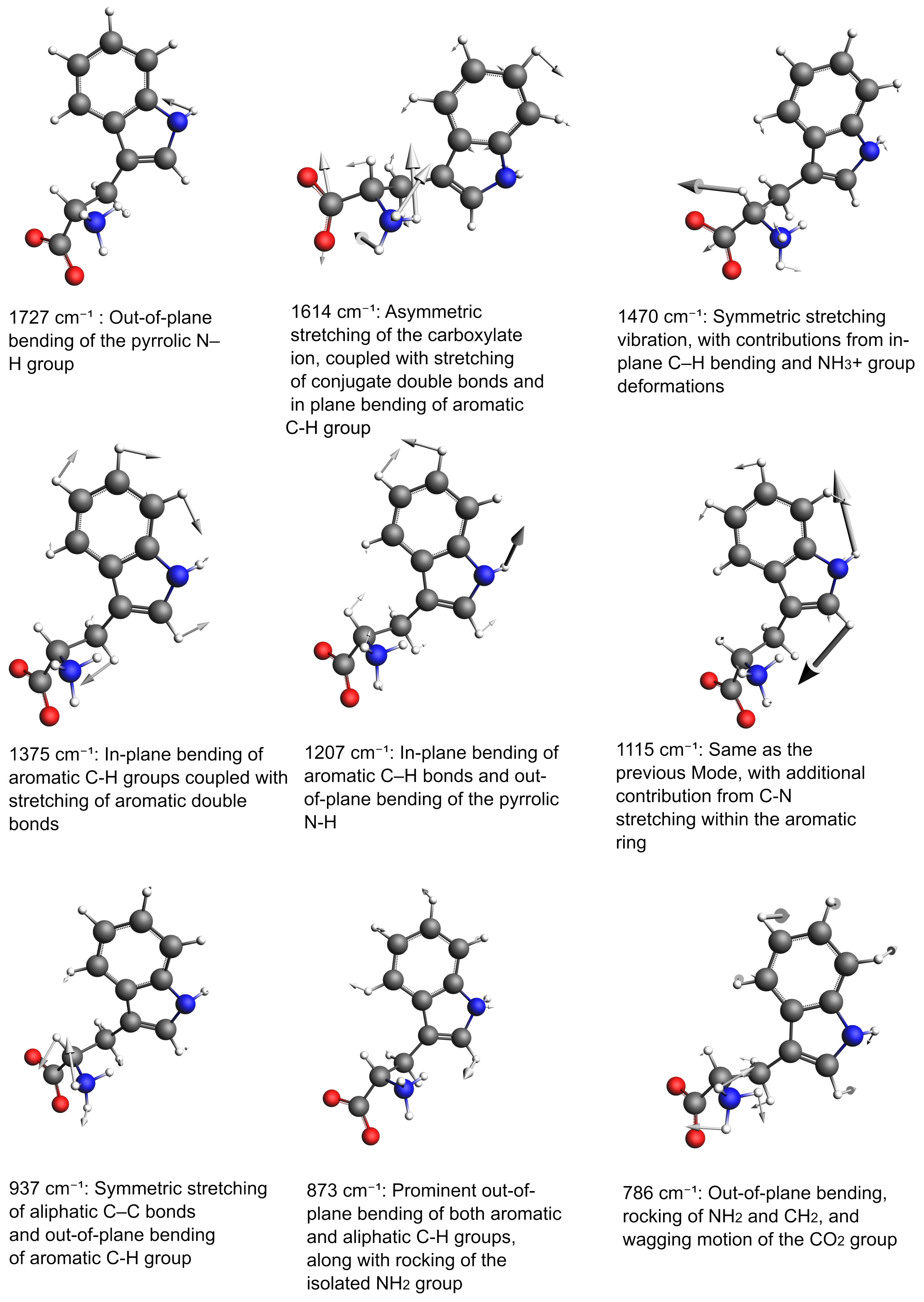

Figure S19: Normal mode analysis of the most representative frame extracted from the MD trajectory, performed using the AMS graphical interface. The calculation is carried out with the QM/FQ model at the B3LYP/TZ2P level of theory.

References

(1) National Center for Biotechnology Information PubChem Compound Summary for CID 6305, Tryptophan. https://pubchem.ncbi.nlm.nih.gov/compound/Tryptophan, 2025; Accessed April 9, 2025.

(2) Wishart Research Group 1H-NMR Spectrum of Tryptophan (HMDB). https://moldb.wishartlab.com/system/documents/files/000/031/283/original/041023_P00_05_HW_1DP_Assigned20121204-87231-1ne9758.png?1354661960, 2025; Accessed April 9, 2025.
